# Fusion and Fission of Cognitive Functions in the Human Parietal Cortex

**DOI:** 10.1093/cercor/bhu198

**Published:** 2014-09-09

**Authors:** Gina F. Humphreys, Matthew A. Lambon Ralph

**Affiliations:** Neuroscience and Aphasia Research Unit (NARU), School of Psychological Sciences, University of Manchester, Brunswick St., ManchesterM13 9PL, UK

**Keywords:** activation likelihood estimation analysis, angular gyrus, attention, episodic retrieval, functional neuroimaging, inferior parietal lobule, intra-parietal sulcus, neuropsychology, numerical tasks, parietal cortex, phonological processing, semantic processing, sentence-level processing, supramarginal gyrus, the default-mode network, tool-related tasks

## Abstract

How is higher cognitive function organized in the human parietal cortex? A century of neuropsychology and 30 years of functional neuroimaging has implicated the parietal lobe in many different verbal and nonverbal cognitive domains. There is little clarity, however, on how these functions are organized, that is, where do these functions coalesce (implying a shared, underpinning neurocomputation) and where do they divide (indicating different underlying neural functions). Until now, there has been no multi-domain synthesis in order to reveal where there is fusion or fission of functions in the parietal cortex. This aim was achieved through a large-scale activation likelihood estimation (ALE) analysis of 386 studies (3952 activation peaks) covering 8 cognitive domains. A tripartite, domain-general neuroanatomical division and 5 principles of cognitive organization were established, and these are discussed with respect to a unified theory of parietal functional organization.

## Introduction

A key goal of cognitive neuroscience is to relate cognitive functions and dysfunctions to underlying neural processing. A common approach, adopted in functional neuroimaging, cognitive neuropsychology and other related neuroscience methods, is to explore 1 specific behavioral domain (e.g., tasks pertaining to attention, episodic memory, language, etc.) and to investigate which brain regions are implicated and how their function varies following changes to important task-related parameters. The considerable productivity of the field is such that significant progress has been made in dissecting both the cognitive machinery and the underlying neural networks in many of these specific behavioral domains. Despite, or perhaps because of this success, 3 key challenges are emerging: 1) like most other natural sciences in the twenty-first century, vast quantities of data are produced, increasing the need for meta-analytic tools to distill the core, reliable findings not only within each specific behavioral domain but also across them; this is because 2) if we step beyond the large literature dedicated to each behavioral domain, then it becomes readily apparent that many neural regions are common to numerous, diverse behavioral activities; generating the conclusion that 3) there is no simple one-to-one mapping between each type of activity and a single underlying brain region. Instead, each behavioral domain maps onto a cortical network, and in reverse, each cortical region seems to play a role in a variety of different cognitive behaviors. Indeed, this neuroscientific maelstrom can sometimes feel like a Shakespearean comedy in which a multitude of characters have a bewildering array of relationships and assume multiple roles. In order to avoid turning comedy to tragedy and simply treating this confusion as some kind of masterpiece, a necessary first step is to map out the overlapping neural bases of different behavioral domains “simultaneously” and then search for a unifying explanation.

**Figure 1.**
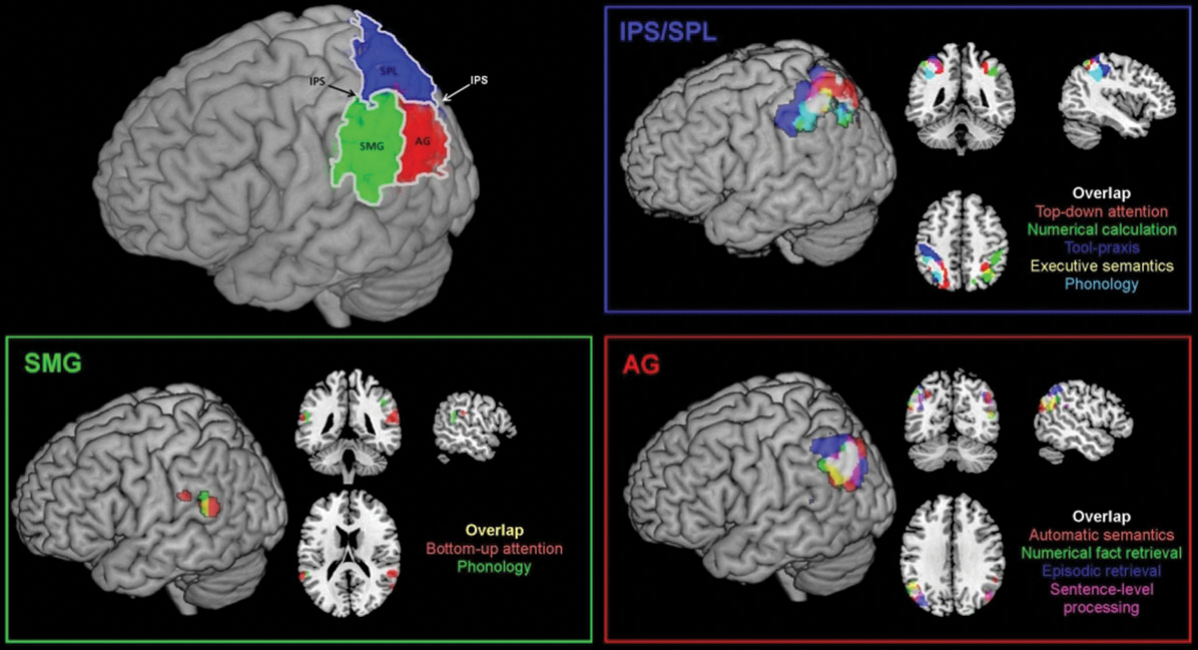
Neuroanatomical location of the parietal cortex and its 3 major divisions; the results from the primary ALE analysis showing differential functional recruitment of IPS/SPL, SMG, and AG. Meta-analysis results were thresholded at FDR correction of *P* < 0.05 and a minimum cluster size of 100 mm³. For clarity, the images are masked to show data from the lateral parietal cortex only (see Supplementary Fig. 3 for whole-brain results).

This investigation focused on the complexity of cognitive functions associated with the (lateral) parietal cortex. This region (Fig. 1) can be segregated into dorsal parietal cortex (DPC) incorporating the intra-parietal sulcus (IPS) and the superior parietal lobule (SPL), and ventral parietal cortex (VPC) containing the supramarginal and angular gyri (SMG and AG) (Nelson et al. 2010; Uddin et al. 2010; Caspers et al. 2011; Mars et al. 2011). These have been further subdivided via their varying cytoarchitectonic properties and structural connectivity patterns (Caspers et al. 2006; Caspers et al. 2008; Uddin et al. 2010; Caspers et al. 2011). These parietal regions have been associated with numerous higher cognitive functions and neuropsychological dysfunction, including attention, episodic memory, semantic cognition, phonology, syntax, praxis, mathematical cognition, theory of mind, etc. For each behavioral domain, there are dedicated overviews and theories for the nature of the underlying representations or cognitive processes that the parietal lobe supports but, until recently, the conundrum posed by packing divergent cognitive activities simultaneously into the parietal cortex has received little attention.

Many theories have been generated in relation to the potential neurocomputations of the parietal cortex. Most (but not all—see below) of these hypotheses try to account for findings from 1 behavioral domain (e.g., semantic processing, episodic memory, number decisions, etc.) and, therefore, tend to be domain specific in nature. Examples of these accounts include the “semantic hypothesis” (Geschwind 1972; Binder et al. 2009) in which the VPC serves as a multimodal store of semantic knowledge and is preferentially engaged for semantic coding of external inputs or self-generated internal content (e.g., default-mode processing). Other versions of this theory suggest that VPC specifically represents “event-semantics” (Binder and Desai 2011). From the area of episodic memory comes the “episodic buffer hypothesis” (Wagner et al. 2005; Vilberg and Rugg 2008) and the “Cortical Binding of Relational Activity (COBRA)” model (Shimamura 2011). Both theories suggest that the VPC is a buffer of multimodal episodic information and thus crucial for episodic memory retrieval. In terms of numerical processing, some researchers have suggested that VPC is a generalized magnitude system, which represents different forms of quantity (time, space, and number) (Walsh 2003). Others have argued that there are 3 parietal circuits for number processing (IPS, AG, and SPL), each serving distinct numerical processes (Dehaene et al. 2003). While each domain-specific theory may account for findings from the behavioral domain in question, it is much less clear how they relate to one another. Although many models suggest that parietal cortex is involved in multimodal processing, they often ascribe different cognitive functions to the same neural areas and they parcellate parietal cortex in different ways.

**Figure 2.**
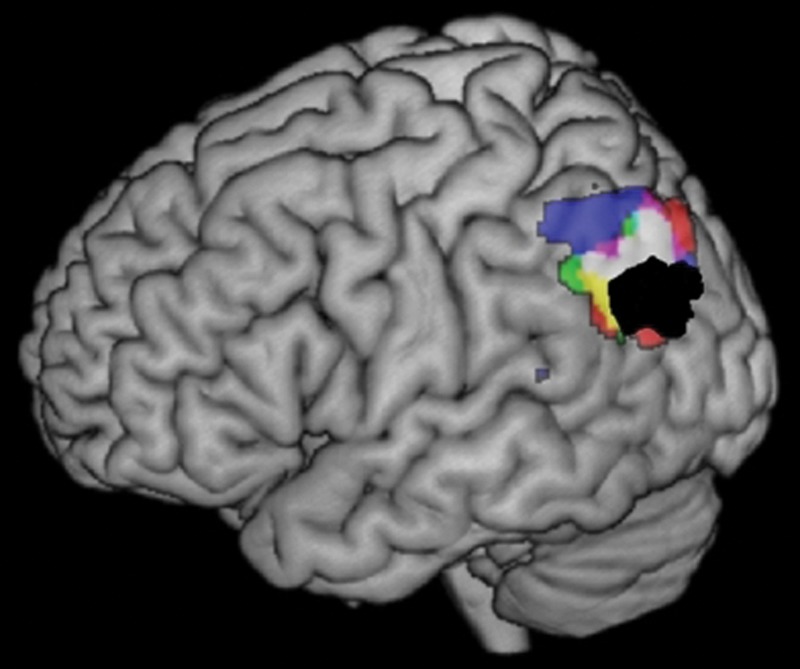
The overlap between the DMN (black) and the AG-related tasks (automatic semantics [red], episodic retrieval [blue], numerical fact retrieval [green], and sentence-level processing [purple]). Meta-analysis results were thresholded at FDR correction of *P* < 0.05 and a minimum cluster size of 100 mm³.

Originally highlighted in Henry Head's seminal work (in which he noticed similarity between semantic, episodic, and attentional-executive deficits in patients with semantic aphasia after parietal wounds: Head 1926), it is only in the very recent literature that the potential overlap and relationship between parietal functions has started to be considered and debated (Cabeza et al. 2012; Nelson et al. 2012) (Fig. 2). These contemporary studies have generated alternative theoretical accounts for how episodic memory and attention might coalesce or dissociate across VPC. For instance, according to the “dual attentional processes hypothesis” (Cabeza et al. 2008), the DPC serves domain-general top-down attention, goal-directed or executively demanding tasks, whereas VPC supports bottom-up attention and automatic task processes. Relatedly, in the “Attention to Memory (AToM) hypothesis” (Cabeza et al. 2012), the VPC supports a bottom-up system that licenses automatic capture of attention by salient external (e.g., loud noises) or internal information (e.g., when a memory “pops” into awareness). These models were developed primarily to account for bottom-up visual attention and episodic memory retrieval data. Thus, while providing important steps toward an overarching theory of parietal function, even these innovative studies are silent on how multiple behavioral domains relate to each other within the parietal cortex (Ciaramelli et al. 2008; Hutchinson et al. 2009; Hutchinson et al. 2014).

This investigation was based, therefore, on a multi-domain synthesis of the considerable functional neuroimaging data that are now available. Through this large-scale meta-analysis, the behavioral–neuroanatomical relationships within the parietal cortex (as well as other regions) were mapped in order to establish 1) which behavioral activities coalesce (indicating shared underlying neurocomputation) and 2) which neuroanatomically dissociate (implying important differences in the neural computations that support each one). The results were used to test 5 potential organizational principles that emerge from the literature (summarized later) as well as specific theories.

1. “Splitting versus lumping neurocognitive functions”: The parietal cortex occupies a large region with multiple cytoarchitectural and connectivity variations. Thus, some researchers have argued that, like a form of neurofunctional marquetry, the parietal cortex is divided into many different domain-specific processing areas each with sharp dissociations in function (Simon et al. 2002; Hutchinson et al. 2009; Nelson et al. 2010; Nelson et al. 2012). Within this “fractionated” view, proposed anatomical-functional pairings include IPS and number processing, SMG with tool-related tasks or phonological processing, and angular gyrus for episodic recollection or semantic processing. In contrast, the “overarching” accounts emphasize greater functional unification reflected in overlapping fMRI activation and co-occurring neuropsychological deficits (Head 1926; Luria 1976; Corbetta and Shulman 2002; Jefferies and Lambon Ralph 2006; Cabeza et al. 2008; Ciaramelli et al. 2008; Duncan 2010; Cabeza et al. 2012; Crittenden and Duncan 2014; Kim 2012; Noonan et al. 2013). The key notion here is that a limited number of underlying neurocomputational processes support multiple behavioral domains.
2. “Dorsal versus ventral parietal cortex”: Classical neuropsychological data clearly suggest a dorsal versus ventral division of function (e.g., ideomotor vs. ideational apraxia, Bálint's vs. Gerstmann's syndromes, etc.: Buxbaum et al. 2006; Vallar 2007). Likewise, functional neuroimaging also suggests a similar division for many different domains (e.g., executive vs. automatic semantic processing, number computations vs. fact recall, familiarity vs. recollection in episodic memory, top-down vs. bottom-up attention, etc.: Corbetta and Shulman 2002; Delazer et al. 2003; Vilberg and Rugg 2008; Whitney et al. 2009; Kim 2010; Noonan et al. 2013). Furthermore, dorsal and ventral areas show different patterns of effective and structural connectivity; dorsal areas form part of a fronto-parietal control system, whereas ventral areas connect with a distributed set of regions associated with the default-mode network (DMN) (Vincent et al. 2008; Spreng et al. 2010; Uddin et al. 2010; Cloutman et al. 2013; Power and Petersen 2013). The dorsal–ventral dimension is orthogonal to the fractionation-unification distinction in that dorsal–ventral differences have been proposed for many individual behavioral domains. Indeed, Bálint's and Gerstmann's syndromes each comprise multiple, superficially unrelated neuropsychological deficits—which might reflect tightly yet separately packed neural functions (i.e., fractionation) or an impairment to a generalized neurocomputation that multiple domains rely upon (i.e., unification).
3. “Anterior versus posterior parietal cortex”: There is evidence for an anterior (SMG)–posterior (AG) dimension. Supramarginal and angular gyri and AG show distinct patterns of connectivity (Uddin et al. 2010; Cloutman et al. 2013) whereas neuropsychological and functional neuroimaging studies suggest a dissociation. In language, SMG tends to be implicated in phonological tasks more than semantics, whereas AG shows the opposite (Price et al. 1997; Devlin et al. 2003). Likewise, in the aphasia literature, conduction aphasia (impaired repetition but intact semantics) is associated with inferior SMG damage (Dronkers et al. 2004; Fridriksson et al. 2010) whereas the opposite pattern (transcortical sensory aphasia) follows from lesions outside of SMG in the watershed territory from IPS through AG to pMTG (Berthier 1999). Outside of the language domain, however, some researchers have argued against strong SMG–AG dissociations, arguing instead for graded functional overlap (Cabeza et al. 2012). Indeed, recent functional connectivity studies find evidence for graded AG–SMG differences rather than strong dissociations (Daselaar et al. 2013).
4. “Positive versus negative activations”: Unlike most other cortical regions, VPC is associated with the DMN (Buckner et al. 2008; Mantini and Vanduffel 2013); this region plus medial frontal and posterior cingulate areas demonstrate “deactivation” when compared with “rest.” Interpretation of deactivation is inherently tricky, and various possible explanations include switching between internal and externally directed cognitive processing (Buckner et al. 2008), or the vibrant semantic-language activity of inner thought, associated with “rest”, is reduced when participants shift into goal-directed non-semantic experiments (Binder et al. 1999). For a complete understanding of parietal functions, it is important to establish the relationship between the DMN and the other behavioral domains. As a first step, we asked the following initial questions: 1) what is the overlap between the parietal component of the DMN and the many behavioral domains associated with the parietal region, 2) do all parietal regions deactivate, and 3) do DMN-parietal regions always deactivate or are positive activations within the same areas sometimes observed?
5. “Laterality”: A final potential dimension of parietal organization reflects left versus right hemispheric differences. Chronic disorders of language or apraxia are associated with left parietal damage whereas long-term visuospatial attention impairments (e.g., neglect) tend to follow from right parietal lesions (Mesulam 1999). This pattern appears to be mirrored in some neuroimaging studies, which have shown right hemisphere activation for attention (Corbetta and Shulman 2002), whereas episodic retrieval and language exhibit left-hemisphere engagement (Vigneau et al. 2006; Hutchinson et al. 2009). While these data suggest that left- and right-hemispheres might serve distinct functions, the strength of these differences is unclear. First, although chronic disorders of visuospatial attention, language, and apraxia are associated with a laterality effect, in the acute phase neglect and aphasia can be observed after left or right lesions (Hier and Kaplan 1980; Kleinman et al. 2007). In addition, few functional neuroimaging studies have conducted formal statistical laterality comparisons. Indeed, even in behavioral domains associated with strong laterality effects, many examples of bilateral activation can be found (Downar et al. 2001; Homae et al. 2002; Herron et al. 2004; Weis et al. 2004; Serences et al. 2005; Mayer et al. 2006; Hutchinson et al. 2014; Vilberg and Rugg 2012).

To assess the principles of parietal organization, we undertook a large-scale, multi-domain meta-analysis based on the considerable functional neuroimaging data that are now available in the literature (see Materials and Methods). We define “behavioral domains” here as a way of categorizing/differentiating different behaviors or higher mental activities (e.g., language behaviors, number decisions, object-use behaviors, etc.). Each of these will be underpinned by a combination of domain-specific and domain-general neurocomputations. If parietal organization reflects a multitude of mutually exclusive domain-specific functions then the meta-analysis results should resemble a form of “neuromarquetry.” Alternatively, if a smaller set of domain-general neurocomputations underpins a wide range of behaviors then the pattern of activations should coalesce into a shared region (or small number of such regions). Briefly, we applied activation likelihood estimation (ALE) analysis (Eickhoff et al. 2009; Turkeltaub et al. 2012) to 3952 activation foci arising from 451 contrasts from 386 studies, covering 8 domains (attention, episodic retrieval, semantic processing, numerical tasks, phonological processing, sentence-level processing, tool-related tasks, and the DMN; see Table 1 for a list of the number of studies and foci for each category and Supplementary Table 1 for all included studies). ALE is a coordinate-based meta-analysis technique that computes the activation likelihood for each voxel and compares this with a null-distribution to determine statistical significance. This method also allows direct comparison of ALE maps to determine statistically significant differences. As noted earlier, some domain-specific reviews have been previously reported. For formal direct comparisons across domains and to link the present unified meta-analysis directly back to the literature, studies were re-selected from key reviews (Binder et al. 2009; Noonan et al. 2013) and supplemented, where necessary, to exceed a minimal sufficiency for meta-analysis of each domain. We also preserved any important subdivisions that have been highlighted in the literature (e.g., top-down vs. bottom-up attention, executive vs. automatic semantic retrieval, numerical calculation vs. numerical fact retrieval). Finally, we examined the extent to which each task and parietal subdivision shows activation/deactivation with respect to “rest.” We focus below on the results for the lateral parietal lobe but the whole-brain results are shown and discussed in Supplementary Material (which are pertinent to on-going debates about prefrontal and posterior temporal functions).

**Table 1.** The number of contrasts and foci, and example contrasts included in the meta-analysis from each domain

Domain	Subclass	Example contrast	*N* contrasts	*N* foci
Semantic memory retrieval	Automatic	Concrete > abstract; words > non-words	38	379
	Top-down	High > low semantic control	35	272
Phonology		Phonology > semantics; phonology > orthography	36	394
Episodic memory retrieval		Old > new judgements; recollection detail correlation	36	400
Attention	Bottom-up	Oddball capture; uncued > cued attention	27	229
	Top-down	Visual search; attentional shift; cue period > target period	24	220
Numerical processing	Fact retrieval	Trained > untrained problems; multiplication > subtraction	18	76
	Calculation	Difficult > easy calculations; untrained > trained problems	24	239
Tools		Tool naming; tool recognition; tool action judgements	48	331
Sentence-level processing		Sentences > word-lists; high syntactic complexity > low complexity; semantically ambiguous sentences > unambiguous sentences	37	263
Default-mode network		All contrasts showing task-related deactivation in the BrainMap database	128	1149
Total			451	3952

## Materials and Methods

### Study Selection

Studies were selected from the behavioral domains of episodic retrieval, semantic cognition (separating automatic semantic activation and “top-down” semantic-based decisions), phonological processing, sentence-level processing, numerical tasks (separating calculation and numerical fact retrieval), tool-related tasks, attention (separating top-down attention and bottom-up attention), and the DMN. This is not an exhaustive list of all behavioral domains that have been associated with the parietal cortex, but they do represent a diverse range of behavioral activities for mapping parietal functions (the relationship between the current results and previous domain-specific meta-analyses for theory of mind tasks and working memory is considered in Discussion). Some other behavioral domains could not be included due to an insufficient number of studies for the ALE method. We aimed for a representative sample of the literature from each domain and, in particular, for the current multi-domain meta-analysis to link directly back to previous seminal reviews or meta-analyses of individual behavioral domains. To this end, studies were re-selected from those included in key reviews or meta-analyses of each behavioral domain. In doing so, this multi-domain meta-analysis was able to preserve the selection criteria and other parameters that are considered highly relevant to researchers working in each subject area (a full description of the method used to define each domain is provided in Supplementary Material) (Cabeza et al. 2008; Ciaramelli et al. 2008; Vilberg and Rugg 2008; Binder et al. 2009; Hutchinson et al. 2009; Ciaramelli et al. 2010; Kim 2010; Arsalidou and Taylor 2011; Cabeza et al. 2011; Cabeza et al. 2012; Noonan et al. 2013). When recent reviews were unavailable (phonological processing), where past reviews provided an insufficient number of studies (bottom-up attention and sentence-level processing) for ALE, or where more up-to-date research is available (automatic semantic retrieval), the study-pool was supplemented with those identified in the Web of Science database using the following search terms: (parietal) AND (episodic, autobiographical, semantic, attention, number, numerical, syntax, syntactic, phonology, phonological). The inclusion criteria were: fMRI or PET studies, studies of healthy young adult participants, no studies of individual differences (e.g., sex, age, and language skills), and studies that reported coordinates of the local maxima in standard space (MNI or Talairach). The DMN was defined using the Brainmap database (http://www.brainmap.org/). Specifically, we selected all studies of healthy participants (irrespective of behavioral domain) in the database that showed task-related deactivations relative to a low-level control condition (either fixation or rest). This is considered a robust and unbiased method for identifying the DMN and has been used successfully elsewhere in the literature (Laird et al. 2009; Smith et al. 2009). Overall, across all domains, 451 contrasts derived from 386 studies met criteria for inclusion, which resulted in a total of 3952 foci. Table 1 shows the number of studies and foci split by behavioral domain, including examples of typical contrasts. Given that this unified meta-analysis deliberately adopted, where possible, studies from previous domain-specific reviews, there was some inevitable variation in selection criteria across (but not within) behavioral domains. Accordingly, additional analyses were conducted to examine the potential influence of variation in selection criteria (e.g., inclusion of studies using ROI-based vs. whole-brain approaches, or contrasts with low-level baseline conditions). These factors were found to have negligible effects (see Supplementary Material).

### ALE Analyses

#### Primary ALE Analyses

The ALE analyses were carried out using GingerAle 2.1 (Laird et al. 2005; Eickhoff et al. 2009). To reveal the networks activated for each of the domains, a primary ALE analysis was carried out on the peaks reported for each domain, separately, using an FDR correction of *P* < 0.05 and a minimum cluster size of 100 mm³. To determine the level of commonality across domains, we examined the overlap from the resultant ALE maps. Figure 1 shows the results of the primary ALE analyses, including the locations of overlap.

#### Subtraction ALE Analyses

Three subtraction analyses were carried out to verify that differences observed in the primary ALE analysis were statistically significant. Visual inspection of the results from the primary individual-domain ALE analyses suggested that there was a difference in the domains that engaged dorsal and ventral parietal areas. This dorsal–ventral difference is in agreement with the second organizational principle highlighted in Introduction. Specifically, we found that certain domains engaged DPC, whereas ventral areas were engaged by a different set of tasks other. Furthermore, within each area, recruitment was highly overlapping across domains but between areas there was no overlap. To test these apparent dorsal–ventral differences formally, the first subtraction analysis examined whether this difference was statistically significant by directly contrasting all “dorsal” tasks with all “ventral” tasks (10 000 permutations; FDR corrected, *P* < 0.05). Secondly, to test for domain-specific processes within dorsal or ventral areas, we directly contrasted each behavioral domain with one another, separately for dorsal and ventral tasks (10 000 permutations; FDR corrected, *P* < 0.01 to correct for multiple comparisons, and also at the less stringent FDR correction, *P* < 0.05, to highlight more subtle effects). Finally, we conducted laterality tests for each domain to investigate any significant differences in ALE values across hemispheres. This was achieved by contrasting each set of foci with that from an identical set but where the *x* coordinate had been inverted (10 000 permutations; FDR corrected, *P* < 0.05).

#### Direction of Activation Relative to Rest

Next, we looked for systematic effects across tasks and neural regions in the tendency for positive or negative activation relative to baseline. Specifically, we noted the direction of relative activation levels (positive or negative) compared with rest/fixation for each foci contributing to the significant clusters in the primary ALE analysis (if this information was reported in the original paper—see Supplementary Material for a summary regarding the proportion of studies that reported this information). These data were collated across foci for each task and neural region to examine any systematic patterns in the data.

#### Non-Parietal Recruitment

Foci from outside parietal cortex were also included in the ALE analysis (FDR correction of *P* < 0.05 and a minimum cluster size of 100 mm³). While the focus in the main paper is primarily on parietal activation, the non-parietal results are relevant to on-going debates about the roles of prefrontal cortex and posterior temporal regions in various behavioral domains (including tool use, language, and semantic functions). Thus, in the Supplementary Material, we also report results from within lateral prefrontal recruitment in linguistic tasks (phonological, top-down semantic, and sentence-level tasks), and the recruitment of posterior temporal areas by top-down semantics and tool-related tasks.

## Results

**Figure 3.**
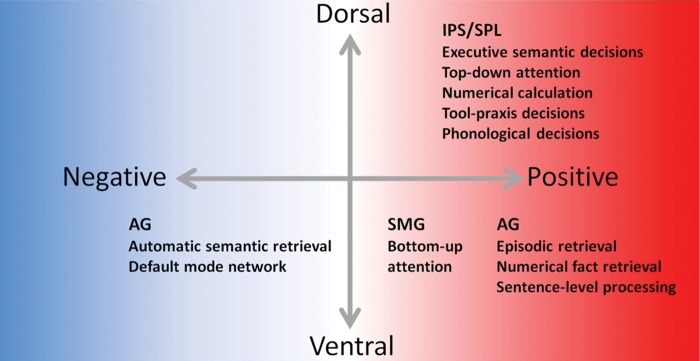
A schematic depiction summarizing the variation in polarity of activation (with respect to rest/fixation) across tasks and neural regions. Tasks associated with IPS/SPL and SMG generate (positive) activations. There is, however, considerable variation within AG ranging from deactivation (DMN and automatic semantic retrieval tasks) through to activation (for the remaining tasks)— see main text for details.

The primary ALE analysis revealed a clear dorsal–ventral distinction, with different task components modulating DPC and VPC areas. Within VPC, a further anterior–posterior difference (SMG vs. AG engagement) was apparent. The results are summarized in Figure 1 (see also Supplementary Table 2 and Fig. 3). A subtraction analysis confirmed that DPC tasks were significantly more likely to engage bilateral IPS and SPL, whereas VPC tasks were significantly more likely to engage the left AG and precuneus (Supplementary Fig. 1).

### Dorsal Parietal Cortex

Several tasks engaged a common IPS/SPL region including top-down attention, numerical calculation, executive semantic decisions, tool-praxis, and phonological decisions. ALE clusters were highly overlapping (maximal MNI overlap: −43, −42, 48 and −36, −45, 49) with some minor variations in cluster extension (anteriorly for tool-praxis decisions into postcentral gyrus; more ventrally for phonology into dorsal SMG; and more posteriorly for top-down attention into posterior SPL—see Table 2).

**Table 2:** The results of pairwise comparisons between the “dorsal parietal” tasks (row task > column task)

	Executive semantic decisions	Phonological decisions	Top-down attention	Numerical calculation	Tool-praxis decisions
Executive semantic decisions	—	N.S.	N.S.	N.S.	N.S.
Phonological decisions	SPL (left)*	—	N.S.	N.S.	IPS/SPL (left)^*^
Top-down attention	SPL (bilateral)*	SPL (bilateral)*	—	SPL (left)*	SPL (bilateral)*
Numerical calculation	IPS/SPL (bilateral)*	IPS/SPL (right)*	N.S.	—	IPS (bilateral)*
Tools	Postcentral gyrus (left)*	N.S.	N.S.	Postcentral gyrus (left)**	—

Note: N.S., not significantly different.

*FDR *P* < 0.01; **FDR *P* < 0.05.

### Ventral Parietal Cortex

An anterior–posterior VPC difference was found, with certain tasks modulating AG and others engaging SMG (see Table 3). Within left AG, overlap (at −48, −64, 34) was revealed for automatic semantics, episodic retrieval, numerical fact retrieval, and sentence-level tasks. This core AG cluster also overlapped with the DMN (see Fig. 3). Beyond these considerable commonalities, there were more minor variations in the cluster extents (see Table 3): Episodic retrieval extended more dorsally than other tasks toward lateral IPS; automatic semantics and sentence-level tasks engaged bilateral AG, whereas episodic and numerical fact retrieval were more left-lateralized. The left ventral SMG was engaged by phonology and bottom-up attention (overlap at −57, −42, 20). The SMG clusters were partially overlapping although phonology was slightly more medial.

**Table 3:** The results of pairwise comparisons between the “ventral parietal” tasks (row task > column task)

	Automatic semantics	Episodic retrieval	Numerical fact retrieval	Sentence-level processing	Phonological processing	Bottom-up attention
Automatic semantics	—	N.S.	N.S.	AG (left)**	AG (bilateral)*	AG (left)*
Episodic retrieval	N.S.	—	N.S.	AG (left)*	AG (left)*	N.S.
Numerical fact retrieval	N.S.	N.S.	—	AG (left)**	AG (left)*	AG (left)*
Sentence-level processing	N.S.	N.S.	N.S.	—	AG (left)*	N.S.
Phonological processing	SMG (left)*	N.S.	N.S.	SMG (left)*	—	SMG (left)*
Bottom-up attention	SMG (bilateral)*	SMG (right)*	SMG (bilateral)*	SMG (right)*	SMG (right)*	—

Note: N.S., not significantly different.

*FDR *P* < 0.01; **FDR *P* < 0.05.

It should be noted here that phonological processing was the only domain to engage both VPC and DPC, which may reflect both top-down and bottom-up components of the phonological tasks. However, it is currently impossible to divide the phonological results because this distinction has not been recognized in the existing literature.

### Polarity of Activation Relative to Rest/Fixation

There were systematic differences in activation versus deactivation across tasks and spatial locations (summarized schematically in Fig. 3). Within DPC, the tendency of each task to show positive activation across contributing foci was 100% in all tasks. The same was true in SMG for bottom-up attention (100% of contributing foci showed positive activation; no data were available for the phonological tasks). In contrast, AG varied systematically depending on task. Specifically, like the variety of studies that were used to define the DMN, automatic semantic retrieval showed a strong tendency for deactivation (89% of contributing foci, which was a significant proportion [binomial test, *P* < 0.05]), whereas for sentence-level tasks and episodic retrieval, activation was almost exclusively positive (100% and 81%, respectively, both significant [binomial test, *P* < 0.05]). Numerical fact retrieval also showed a tendency for positive activation (67% of foci, although not reaching significance; *P* > 0.05). Interestingly, the tasks that have been contrasted most often with numerical fact retrieval (difficult calculations of untrained or difficult mathematical problems) deactivate AG (100% of cases). In summary, despite the high neuroanatomical overlap across AG tasks, the polarity of AG activation varies dramatically. This novel fact suggests an important organization principle for the functional role of the AG versus other parietal regions.

### Laterality Analysis

Three patterns were revealed by the ALE analysis (all FDR *P* < 0.05): 1) left-only for executive semantics, phonological processing, and tool-praxis decisions; 2) bilateral (left > right) for automatic semantic retrieval, episodic retrieval; 3) bilateral (left = right) was found for attention (bottom-up and top-down attention), numerical calculation, and sentence-level tasks.

## Discussion

What does this multi-domain meta-analysis tell us about the 5 potential principles of parietal organization?

1. “Lumping versus splitting of functions”: 1) the extreme positions of either highly “fractionated” models or complete functional unification are unlikely to be correct. Instead, the tasks appear to coalesce into a triad of regions; 2) the fates of these areas are not governed by domain but rather the cognitive components required by each task. Given the diversity of tasks that engage the 3 same areas, an explanation based on domain-general processing systems seems the most potent.
2. “Dorsal versus ventral parietal regions”: In keeping with classical neuropsychology and functional neuroimaging (Corbetta and Shulman 2002; Delazer et al. 2003; Buxbaum et al. 2006; Vallar 2007; Vilberg and Rugg 2008; Whitney et al. 2009; Kim 2010; Noonan et al. 2013), the data indicate a clear and reliable split between IPS/IPL and AG/SMG. DPC is associated with both verbal and nonverbal executively demanding tasks. This is consistent with the notion that this region is a part of a multi-domain demand/control system (Duncan 2010; Whitney et al. 2012). VPC is linked to verbal and nonverbal tasks which tap into more “automatic” processes. This result fits with a domain-general version of the dual-attention processing model (Corbetta and Shulman 2002; Cabeza et al. 2008), which proposes that DPC is implicated in “any” goal-directed, executively demanding task. In contrast, VPC is engaged in all tasks that involve more automatic and stimulus-driven processes. This meta-analysis demonstrates that the dorsal “top-down/executive” versus ventral “bottom-up/automatic” distinction holds across multiple domains beyond attention and episodic memory.
3. “Anterior versus posterior areas”: Within VPC, the data indicate a secondary split such that phonology and “bottom-up” attention are associated with ventral SMG/temporoparietal junction. The remaining tasks and DMN are linked to the AG. Thus, beyond the dorsal–ventral distinction, the current data suggest an additional anterior–posterior VPC gradient. This dimension was not considered in the original versions of the dual-processing model, but it has been highlighted by direct contrasts of attention and episodic memory (Hutchinson et al. 2009; Hutchinson et al. 2014) and has been integrated into graded versions of the dual-processing model (Daselaar et al. 2013).
  Dimensions 1–3 indicate the presence of 3 functionally dissociable, multi-domain parietal networks: IPS/SPL, AG and SMG. This finding fits with resting-state functional connectivity and tractography studies, in which the same areas demonstrate different connectivity patterns: The AG forms part of the DMN alongside the medial prefrontal cortex, posterior cingulate cortex, and medial temporal lobes; the SMG forms part of a cingulo-opercular system involving the lateral prefrontal cortex, anterior cingulate cortex, and frontal operculum/insula; and IPS/SPL is part of a fronto-parietal control system connected with dorsolateral and medial prefrontal cortex (supplementary motor area), MT+ and the frontal eye fields (Vincent et al. 2008; Spreng et al. 2010; Uddin et al. 2010; Cloutman et al. 2013; Power and Petersen 2013).
4. “Activation polarity”: A new functional dimension was revealed by the meta-analysis. In IPL/SPL and SMG, activations were positive across all tasks (> rest/fixation). In contrast, the AG appears to exhibit a full spectrum from deactivation for the many tasks associated with deactivation in the DMN as well as “automatic” semantic processes, through to positive activation for sentence-level tasks, episodic and number-fact retrieval.
5. “Laterality”: The data indicate graded differences across hemispheres which do not align neatly with a verbal versus nonverbal distinction. The only tasks to be associated with a left-only distribution were phonological, executive semantics and tool-praxis decisions. This is consistent with the observation that chronic (phonological or semantic) aphasia and apraxia are associated with left-hemisphere damage (Buxbaum et al. 2006; Jefferies and Lambon Ralph 2006). The remaining tasks had a bilateral distribution with only automatic semantic and episodic retrieval exhibiting a graded left > right pattern (which might reflect the fact that these functions have been predominantly probed through the verbal domain in fMRI studies).

### Reevaluating Theories of Parietal Function

The extant theories vary in how well they map onto these 5 dimensions of parietal organization. Many of these hypotheses were created to explain various “domain-specific” results, including number, space and time (Dehaene et al. 2003; Walsh 2003); semantic representation (Geschwind 1972; Binder et al. 2009; Binder and Desai 2011); or episodic recollection (Wagner et al. 2005; Vilberg and Rugg 2008; Shimamura 2011). Thus, unsurprisingly, they are relatively silent when it comes to explaining the broader multi-domain results. Given the tripartite domain-general overlap of parietal functions appears to be inconsistent with the “neuromarquetry” hypothesis (that each domain reflects modular processing within isolated parietal subregions), we should, instead, consider how well current theories explain the pattern of data within and across the regional triumvirate.

#### Dorsal Parietal Cortex

Several researchers have suggested that DPC forms part of a domain-general fronto-parietal executive control system (Corbetta and Shulman 2002; Cabeza 2008; Duncan 2010). Consistent with this proposal, we found a high degree of spatial overlap within IPS/SPL for a variety of tasks including top-down attention, numerical calculation, executive semantics, phonological tasks, and tool-praxis decisions. Given such a wide variety of tasks and domains, it seems likely that this region supports a general processing system. As noted earlier, the common factor is that these tasks are non-automatic, goal-directed and have high-executive demands. While obvious for top-down attention and numerical calculation, the executive demands in the other tasks may not be immediately clear. The executive semantic and phonological tasks require careful and precise decisions or comparisons across multiple stimuli. Indeed, a recent meta-analysis that contrasted high- versus low-demand semantic tasks highlighted IPS as a key component (along with inferior PFC and pMTG: Noonan et al. 2013). Likewise, the tool-praxis tasks have the same high-demand decision and working memory requirements, reflected in long decision times (Kellenbach et al. 2003; Ishibashi et al. 2011).

The DPC results are less consistent with the strong “fractionated” view (Simon et al. 2002; Nelson et al. 2010; Nelson et al. 2012). It should be noted, however, that there were some graded differences in the extension of the overlapping IPS clusters to other neighboring areas—although these differences were not absolute. Specifically, the tool-praxis cluster extended into postcentral gyrus, which would be consistent with a role of somatosensory areas in praxis. Secondly, top-down attention exhibited more extensive recruitment of SPL, extending medially and posteriorly toward the precuneus. The role of dorsal–medial SPL is not currently clear, and activation of this region has been reported for other domains, such as tasks involving low-confidence episodic retrieval. Consequently, this region is also not solely associated with top-down attention (Hutchinson et al. 2014). Our data suggest that there may be “graded overlap”— parietal cortex is organized along domain-general lines but that there may be second-order graded differences based on variations in connectivity (e.g., tool-praxis connects with a somatosensory network in postcentral gyrus).

#### Ventral Parietal Cortex

The majority of VPC-associated tasks (but not all, e.g., sentence-level tasks, see below) are more automatic, stimulus-driven and have lower executive demands (i.e., the computed contrasts tended to reflect a comparison of lower > higher executive demands, such as trained > untrained mathematical problems and concrete > abstract semantics). This contrapositive relationship between DPC and VPC is consistent with the thrust of the domain-general, dual-attention hypothesis or the internal versus external processing hypothesis for the DMN (Corbetta and Shulman 2002; Buckner et al. 2008) and is less consistent with any of the domain-specific approaches. The other principles of parietal organization (anterior vs. posterior; polarity of activation; laterality) are not obviously captured, however, by these domain-general proposals. Instead of a simple contrapositive dance, DPC and VPC seem to play neurocomputational counterpoint in which the nature of VPC function varies across task not only in terms of which subregion (SMG vs. AG, unilateral vs. bilateral) contributes but also whether the AG is activated or deactivated. Some, but not all, of these features might be captured by the AToM hypothesis, which argues that VPC bottom-up attention is triggered not only by highly salient external events (e.g., flashing lights, noises) but also by highly salient internal events such as when a remembered item “pops” into awareness (Cabeza et al. 2008). There are at least 3 aspects that remain a puzzle. First, decisions to externally presented sentences positively activate AG, whereas word tasks, for example, do not. Secondly, expectancies are often broken (a saliency signal) in the many verbal and nonverbal experimental tasks, which more commonly deactivate AG and activate DPC. Finally, bottom-up attention and episodic memory engage different VPC regions (vSMG vs. AG, respectively) as shown not only in this multi-domain meta-analysis but also in studies utilizing a within-subjects direct comparison (Hutchinson et al. 2009), although recent incarnations of AToM have suggested more graded functional and connectivity differences across SMG and AG (Daselaar et al. 2013).

Many domain-specific hypotheses have been formulated around processing in the AG or SMG. Thus, a second explanatory approach could be generated from the assumption that DPC supports a domain-general executive system whereas domain-specific processing occurs in VPC. No one domain-specific theory, however, appears to be able to explain the multi-dimensional counterpoint pattern of VPC data. Key challenges are found in explaining the overlap of functions in ventral SMG and the separate set in AG, as well as its negative-to-positive activation polarity. First, we need an explanation for why left vSMG acts simultaneously as a phonological buffer/sensory-motor interface for speech (Baddeley 2003; Hickok and Poeppel 2007; Rauschecker and Scott 2009), and bilaterally, it supports bottom-up attention (Corbetta et al. 2008). Secondly, various domain-general roles have been ascribed to AG. Geschwind (1972) suggested that AG might support the formation of multimodal semantic representations, and this idea is prominent in the functional neuroimaging literature, driven by the fact that AG shows sensitivity to semantic factors (e.g., concrete > abstract, words > non-words: Binder et al. 2009). An elegant extension to this notion has already been proposed for the DMN in that during “rest,” participants engage in inner thought and speech which necessitate the semantic network (Binder et al. 1999). AG deactivation would follow when switching from semantically busy “rest” to a non-semantic/language experimental task. The semantic hypothesis might also be extended to AG sentence-level and episodic memory activation given that both are semantically rich stimuli or if the AG representation reflects “event-semantics”(Binder and Desai 2011). There appears, however, to be various significant challenges for this hypothesis to explain. First, posterior semantic aphasic patients (whose lesions overlap with VPC) do not seem to have degraded semantic representations, unlike those with anterior temporal damage such as semantic dementia. Instead, SA patients have impairment in flexible, context- and time-appropriate use of semantic and other non-semantic types of information (Head 1926; Jefferies and Lambon Ralph 2006). Secondly, although not observed in the original paper on a semantic explanation for the DMN (Binder et al. 1999), the vast majority of semantic neuroimaging studies generate AG deactivation and the repeatedly observed difference for easy > hard semantic contrasts reflects deactivation disparities (i.e., greater deactivation for the more challenging condition), which can be flipped for the same stimuli if the task instructions reverse their difficulty (Pexman et al. 2007). Finally, along the AG activation polarity dimension, semantic and other tasks used to define the DMN generate deactivation whereas episodic memory, number-fact retrieval, and sentence-level processing engender “positive” AG activations. Thus, it seems unlikely that the positive AG activations in these domains reflect semantic content.

An alternative approach suggests that either the AG supports temporary storage of multimodal episodic information, which acts as an interface between executive processes and stored episodic representations (Wagner et al. 2005; Vilberg and Rugg 2008), or the AG supports a long-term, multimodal episodic store (the COBRA model: Shimamura 2011). These hypotheses are motivated to explain VPC data for episodic tasks, specifically, and so, it is less obvious how they can be extended to the other dimensions of parietal organization. For the DMN and semantic deactivations, one would have to assume that “rest” is primarily filled with episodic activity but it does not explain why there are differential, difficulty-related deactivations. In addition, the positive AG activation for number-fact retrieval or sentence-level processes is also puzzling for this approach unless one assumes that these domains utilize the same short-term buffer.

### Parietal Unified Connectivity-Biased Computation (PUCC)

This penultimate section sketches out a new unified framework that attempts to place a girdle around the 5 dimensions of parietal organization. It adopts existing ideas from the domain-general and domain-specific theories and assimilates them with insights from connectivity information and computational modeling. Parietal unified connectivity-biased computation (PUCC) is founded on 2 sets of assumptions. The first is that ventral brain pathways extract information about content (e.g., semantics) that can be generalized across time and context (Lambon Ralph et al. 2010; Lambon Ralph 2014), whereas the opposite is true for the dorsal pathways (Ueno et al. 2011; Bornkessel-Schlesewsky and Schlesewsky 2013). By buffering time-, context-, and space-varying inputs, it is possible for parietal systems to become sensitive to content-invariant structures/schemata. Effective continuous verbal (e.g., speech) and nonverbal behaviors (e.g., sequential object use) require careful synchronization of planned steps with information about the current state of the external and internal worlds. This information, however, arrives through different internal and external input channels and is ephemeral, necessitating a multimodal convergent buffer. Although in classical Broadbentian modular frameworks, there is a separation of temporary storage from long-term memory, 1 school of contemporary computational models suggest that repeated buffering can lead to long-term statistical learning. Although there is an on-going debate in the computational modeling literature (e.g., Seidenberg et al. 2002; Marcus and Berent 2003), there has been a long history and a wide range of parallel distributed processing models for language, short-term memory, and sequential object use, which have demonstrated that systems designed to buffer and reproduce sequential inputs also become sensitive to the long-term, time-based statistical structures present in the data (McClelland et al. 1989; Botvinick and Plaut 2004, 2006; Ueno et al. 2011). The second assumption is that DPC and VPC are tertiary association cortices with multimodal inputs but with graded variations in long-range connectivity: DPC has preferential connectivity to lateral prefrontal regions, whereas VPC has varying connections to different temporal and inferior prefrontal areas (Vincent et al. 2008; Spreng et al. 2010; Uddin et al. 2010; Cloutman et al. 2013; Power and Petersen 2013). Computational models have shown that even if local (unit) computations are identical (e.g., time-based buffering), there are emergent variations in function across a continuous computational layer as a consequence of differential long-range connectivity (Plaut 2002).

“Principle 1—tripartite division”: follows directly from these 2 sets of assumptions. Multimodal buffering and emergent time/space/context statistical extraction are required across many different verbal and nonverbal activities and thus domain-general patterns emerge. The tripartite division emerges as a consequence of the different long-range connectivity patterns.
“Principle 2—dorsal versus ventral”: By connecting with lateral prefrontal cortex, DPC forms the domain-general multi-demand network (Duncan 2010). Most notions of executive control and attention require a combination of selection and manipulation processes that interface with internally buffered information, which might be reflected in prefrontal regions sending top-down signals to DPC, as recently demonstrated in primate electrophysiological studies (Crowe et al. 2013). Ventral parietal cortex might also buffer multimodal input but without the influence of prefrontal goal-directed cognition. In Baddeleyean working memory terminology, the DPC would represent a multimodal “episodic buffer,” which requires direct interaction with “executive” systems, whereas VPC would be an automatic, multimodal “slave” buffer. Recent fMRI time-windowed correlation analysis of continuous visual versus auditory stories is consistent with this multimodal buffer function for VPC (Lerner et al. 2011). The dual-attention model would emerge from this organization in that “bottom-up” attention reflects the active contents of the automatic multimodal VPC buffer and orientation would be triggered intrinsically by any elements that are inherently important as signaled by valence, meaning, or incoherence with the current context (Goulden et al. 2014). Following the computational principles noted earlier, repeated buffering of time- or space-varying inputs will result in extraction of content-invariant statistical structures. Within the verbal domain, we generally refer to these as phonology and syntax (reflecting extraction of statistics over short and long time-windows) and in the nonverbal domain as schemata (e.g., for sequential object use). Following the assumption of the AToM theory of magnitude (Walsh 2003), it is easy to extend this same representation-from-buffering notion to number and space in that all representation types are content-invariant and generalizable.
  To clarify our working hypothesis, we propose that parietal organization is not split by “domain”/task, rather it reflects the differing neurocomputations needed to meet the demands of every task. Thus, for the same task, different parts of the parietal cortex will be engaged and this pattern could change during a task. We propose that both VPC and DPC might act as a buffer of activated information but the precise neurocomputation varies depending on connectivity—resulting in dorsal areas acting as an executive buffer and ventral areas as an automatic buffer. These 2 networks are, however, closely inter-related. For example, during a well-rehearsed activity, VPC will automatically buffer the multimodal on-going information, which will be useful for timing and coordinating steps in sequential and multimodal activities. During the activity, however, if a stimulus or string of stimuli enter the automatic buffer that have high valence or violate expectations (e.g., the pattern of words is an atypical syntactical pattern or sequence of events), then this will trigger a saliency signal, which in turn would activate top-down processes. Thus, in this situation, the same task will engage both VPC and DPC depending on the demands of the situation. This is akin to and inspired in part by the notion of a “circuit-breaker” proposed by Corbetta and Shulman (2002) in which VPC acts as an alerting system for DPC. It is probably worth noting here that many fMRI experiments are designed to explore a certain activity/domain and, in order to be able to measure behavior explicitly (e.g., with RT and error rates), the participant is required to complete a novel task that requires monitoring and/or a decision (i.e., executive processing) and, thus, many studies commonly engage and activate the DPC. In this context, we should note that is potentially puzzling that sentence-level tasks, sometimes with complex syntax, were found to recruit only VPC regions in this meta-analysis. It may be explained, however, by certain properties of the studies many of which involved passive tasks (passive listening or reading), and hence, this may have minimized the top-down processing demands since participants were not required to make a decision about the item. The prediction, here, is that if a decision on such items was also required then the DPC would become engaged.
“Principle 3—anterior versus posterior”: VPC subdivisions probably also emerge from differential connectivity of SMG versus AG to various temporal and frontoinsular regions (Uddin et al. 2010; Cloutman et al. 2013). AG involvement in multiple verbal and nonverbal domains follows directly from its multimodal connectivity. The pairing of vSMG with phonology is a direct reflection of its intermediary role between auditory input and motor-speech (Rauschecker and Scott 2009; Ueno et al. 2011; Cloutman et al. 2013). The overlap between phonology and attention is a puzzle—although, significantly, a similar overlap has been found in a recent meta-analysis using a very different methodology, although this time in the right hemisphere (the left hemisphere was not included in that study) (Bzdok et al. 2013). This could result from 4 factors: 1) possible vSMG connectivity with posterior ventrolateral regions that code visual motion, 2) the fact that attention in everyday life is triggered by sounds, and thus vision and sound need to be synchronized, 3) the shift from SMG to AG tasks seems to mirror a graded change in time granularity, such that domains requiring computation over short (SMG: phonology and attention) and long time-windows (AG: syntax, schemata, number, etc.) vary along this anatomical axis, 4) the shift reflects a change in externally directed attention (SMG) to internally directed attention (AG), as suggested by existing theories (Bzdok et al. 2013).
“Principle 4—polarity of activation”: Taking each region in turn: 1) as a part of the multi-demand system, DPC activity will rise in line with task difficulty, 2) VPC will activate when tasks tap into long-term, buffer-extracted, content-invariant statistical structures (i.e., episodic recollection, phonology, syntax, time-, space-, or number-schemata), but for executively demanding tasks that do not require this information, continued automatic buffering of related and unrelated, external events in VPC is disruptive to targeted, goal-directed decisions supported by DPC. Performance can be improved, therefore, by inhibiting the VPC “slave” buffering (a cortical version of blocking ears and closing eyes in order to “concentrate”) but, in order to balance task performance against awareness to on-going external events, VPC inhibition is titrated against task difficulty, thus explaining why VPC deactivation is greatest for the most challenging tasks or stimuli.
“Principle 5—laterality”: Most tasks recruit DPC/VPC bilaterally except phonology, semantics, and object praxis. Thus, a verbal/nonverbal distinction cannot underpin the limited laterality differences. Instead, the left-sided pattern for phonology and praxis could reflect connectivity to prefrontal motor control areas which, for speech and fine-motor movements, is strongly left-lateralized (Thiel et al. 2001; Blank and Wise 2002) whereas left-sided semantics might reflect the predominant use of written word stimuli to probe performance (Marinkovic et al. 2003).

### Relating PUCC to Other Cognitive Domains and Neuropsychological Findings

As noted in the section Materials and Methods, this overarching meta-analysis did not contain an exhaustive coverage of all cognitive domains that engage parietal cortex. Some of these have an insufficient number of studies for ALE analysis, but 2 domains have been carefully studied previously (working memory and theory of mind) and these independent meta-analyses provide further support for the PUCC hypothesis. A meta-analysis of working memory studies (n-back tasks) showed that DPC is reliably activated (Owen et al. 2005), the locus of which overlaps with the DPC component identified in this study. Through the investigation of fMRI and neuropsychological deficits, there is increasing convergent evidence for the notion that DPC is involved in the storage and manipulation of items held in working memory (Jonides et al. 1998; Koenigs et al. 2009). This proposal is fully consistent with the assumptions of PUCC in which parietal cortex is a temporary buffer of activated information, and DPC is specifically involved in goal-directed/executively demanding task processes, such as working memory. With regard to theory of mind, a recent meta-analysis identified consistent AG recruitment (Spreng et al. 2009), the locus of which overlaps with the VPC component in the current study. In many respects, theory of mind tasks (which typically involve considering an alternative point of view to the one that is dominant) parallel those from syntactic complexity, also considered in the current study; a common experiment design used in both behavioral domains involves a violation of the expected sequence of events.

Although this discussion has inevitably focused primarily upon the functional neuroimaging data, we finish by considering how the PUCC framework fits with the large and long-standing neuropsychological literature. Indeed, as noted earlier, observations from classical and contemporary neuropsychological studies were part of the motivation for this unified meta-analysis and have inspired some of the principles. Although it is only relatively recently that functional neuroimaging studies have explored the potential parietal overlap across different behavioral domains, this notion has a long history in neuropsychology; Bálint's and Gerstmann's syndromes, for example, were denoted as “syndromes” because the same underlying lesion caused a constellation of co-occurring symptoms across a range of tasks/behavioral domains (cf. Principle 1—coalescence of behavioral domains). With regard to Principle 2 (dorsal vs. ventral parietal functions), again the neuropsychological findings would appear to be consistent with the general patterns found in this unified meta-analysis. For example, Bálint's syndrome (resulting from DPC damage) is associated with impaired object-directed reaching (optic ataxia) and intentional eye-movements (oculomotor apraxia) i.e., tasks that are goal-directed. Contemporary lesion-based neuropsychology has also highlighted that DPC damage is associated with working memory (i.e., executively demanding) deficits rather than simple span or recall ability (Koenigs et al. 2009) Furthermore, our data on DPC mirror current debates in the apraxia literature, where some researchers have suggested that damage to dorsal parietal areas is related to executive deficits in problem-solving, perhaps in addition to the more traditional accounts of motor planning (Goldenberg 2009). With regard to VPC, again a number of neuropsychological deficits have been associated with damage to this region, which contrast with DPC-related impairments. For instance, Gerstmann's syndrome is associated with deficits in more automated tasks such as digit processing (dyscalculia) and spelling (dysgraphia) (Vallar 2007). Likewise, semantic aphasia was classically associated with parietal damage (Head 1926; Luria 1976), and more recent studies have identified co-occurrence of Gerstmann's syndrome and semantic aphasia in the same patients (Hier et al. 1980; Ardila et al. 2000) and of verbal and nonverbal (tool use) impairments in semantic aphasia (Corbett, Jefferies, Ehsan, et al. 2009; Corbett, Jefferies and Lambon Ralph 2009) Intriguingly, Head (1926) and later Goldstein (1936) and Luria (1976) considered that these patients' semantic impairment may be part of a more general deficit in processing abstracted symbolic representations (which might fit with VPC extraction of content-invariant statistics as proposed under the PUCC hypothesis). AG damage has also been related to deficits in short-term memory for sentences (Dronkers et al. 2004), consistent with the notion of a buffering system. Again there appears to be an overlap between patients with “semantic buffer” impairments and (mild) semantic aphasia (Hoffman et al. 2011, 2012). Consistent with the loss or degradation of automatic “slave” short-term buffering, such patients can demonstrate a very striking form of disordered sentence recall in which long, scrambled sentences cannot be veridically recalled but instead the patients reorder the information back into semantically and syntactically correct sentences (Hoffman et al. 2011, 2012).

The separation of SMG and AG functions (Principle 3) is fairly well-established in the neuropsychological literature. As noted in Introduction, within the verbal domain, phonological deficits are associated with lesions to or stimulation of SMG (Sakurai et al. 1998; Hartwigsen et al. 2010) whereas semantic impairments are linked to more extra-sylvian regions (from pMTG through to AG [Chertkow et al. 1997; Berthier 1999; Jefferies and Lambon Ralph 2006; Robson et al. 2012]). The overlap between semantic impairments and other deficits has a variety of positive data (see above). In contrast, the novel finding of an overlap between “bottom-up” attention and phonological processing is, we believe, untested in the neuropsychological literature—though the more clearly bilateral nature of attention (than phonology) may itself generate behavioral differences in unilateral patients (one might expect performance on bottom-up attention tasks to be more robust due to bilateral involvement, though see Hartwigsen et al. 2010). In comparison with Principle 3, it is more challenging to consider the neuropsychological corollaries of the varying polarity of AG activation (Principle 4) as this (like the DMN) is a phenomenon that pertains, perhaps specifically, to functional neuroimaging. Translation into meaningful neuropsychological investigations will require further elaboration of the key VPC neurocomputations and an understanding of the basis of their complex de-/activation patterns.

Finally, Principle 5 fits fairly readily and consistently with the neuropsychological literature. As noted in Introduction, chronic aphasia and apraxia are associated with left-hemisphere lesions and these behavioral domains exhibited a left-sided distribution of imaging peaks in this unified meta-analysis. Other domains appear to be bilateral in nature, and this predicts that clear, clinically notable neuropsychological deficits should be associated with biparietal diseases (as they are in Alzheimer's disease, posterior cortical atrophy, and corticobasal degeneration—though the latter often has an asymmetric clinical presentation early in the disorder [Hodges et al. 1999; Perry and Hodges 1999; Crutch et al. 2012]). One potential inconsistency relates to chronic disorders of visuospatial attention, which was found to be bilateral in form in this unified meta-analysis yet is associated with right hemisphere lesions in the neuropsychological literature (Molenberghs et al. 2012). Two possible explanations can be considered here. The first is that much less is known about visuospatial processing in left parietal patients because aphasia is a common exclusion criteria and thus visuospatial deficits might be more common than currently thought. Secondly, direction of spatial attention may be a key factor (given that visuospatial neglect is observed in the hemifield contralateral to the lesion location). Unfortunately, we were unable to investigate this potentially important dimension in this unified meta-analysis due to insufficient imaging studies.

As a final postscript to this neuropsychological discussion, we should note that there are intrinsic challenges in relating the neuropsychological and neuroimaging literatures. On the one hand, functional activations do not prove the necessity of a region (Price and Friston 2002). On the other hand, directly mapping patient data onto neuroimaging findings is inherently challenging given that patient lesions are often large and do not respect functional–anatomical boundaries, such as the dorsal–ventral or anterior–posterior distinction. Furthermore, almost all lesions (across different neurological etiologies) not only affect gray matter but also penetrate into the underlying white matter thus potentially (but not necessarily) disrupting connections between areas. As a result, neuropsychological data may be complex or even run counter to expectations. For example, in the context of DPC versus VPC functions, contemporary in vivo tractography and dissection studies have shown that DPC receives major connections from prefrontal and temporal areas via the parietal branches of the inferior occipito-frontal fasciculus and inferior longitudinal fasciculus, which course under the VPC (Schmahmann et al. 2007; Martino et al. 2010; Duffau et al. 2013). Consequently, it is entirely possible that VPC-focused damage might sever the connections to DPC, and thus, the resultant neuropsychological pattern could resemble that expected for DPC lesions (i.e., generalized executive impairments arising from remote disconnection). While these challenges are an inherent feature of neuropsychology, when combined with modern high-resolution neuroimaging, some of these conundrums may be solved in future investigations through careful, large-scale voxel-lesion symptom mapping as well as in vivo tractography.

## Summary

A meta-analysis of 8 cognitive domains found a tripartite domain-general organization of parietal function. Five principles of parietal organization were revealed, which cannot be captured in their entirety within existing theoretical accounts of parietal function. A new framework is proposed (Parietal Unified Connectivity-biased Computation: PUCC), which assimilates many existing notions and combines them with computational and connectivity insights. Specifically, out of domain-general multimodal buffering, a tripartite parietal division emerges as a consequence of differential long-range connectivity. Furthermore, long-term statistical extraction from repeated buffering generates the various verbal and nonverbal representations that are associated with the parietal lobe.

## Supplementary Material

Supplementary material can be found at: http://www.cercor.oxfordjournals.org/.

## Funding

This study was supported by a Medical Research Council (UK) grant to MALR (MR/J004146/1). Funding to pay the Open Access publication charges for this article was provided by a UK Research Councils (RCUK) award to the University of Manchester.

## Supplementary Material

Supplementary Data
