# Supplementary material for: Fusion and Fission of Cognitive Functions in the Human Parietal Cortex
Source: Cereb Cortex. 2014 Sep 9;25(10):3547–60. doi: 10.1093/cercor/bhu198 (PMC4585503; doi:10.1093/cercor/bhu198)
Supplement: Supplementary Data [file supp_25_10_3547__index.html]

Fusion and Fission of Cognitive Functions in the Human Parietal Cortex — Fusion and Fission of Cognitive Functions in the Human Parietal Cortex — Supplementary Data 

# Fusion and Fission of Cognitive Functions in the Human Parietal Cortex

## Supplementary Data

Supplementary Data

**Files in this Data Supplement:**

- Supplementary Table 1 - pdf file
- Supplementary Data - Docx file
- Supplementary Figure 1 - jpg file
- Supplementary Figure 2 - jpg file
- Supplementary Figure 3 - jpg file
