# Supplementary material for: Fusion and Fission of Cognitive Functions in the Human Parietal Cortex
Source: Cereb Cortex. 2014 Sep 9;25(10):3547–60. doi: 10.1093/cercor/bhu198 (PMC4585503; doi:10.1093/cercor/bhu198)
Supplement: Supplementary Data [file supp_bhu198_bhu198supp_table1.pdf]

Domain	Study	N	Stimuli	Task	Contrast	X MNI	Y MNI	Z MNI
**Automatic semantics**	Assaf et al., 2006	18	Words	Semantic decision	Related > unrelated	-12	46	-2
						-8	-36	38
						-40	-47	50
						-66	-55	-4
	Bedny et al., 2006	13	Words	Semantic decision	Concrete > Abstract	-27	-78	39
						-21	18	54
						45	-69	24
	Binder et al., 2005a	24	Words	Lexical decision	Concrete > abstract	-37	-74	26
						52	-58	22
						54	-48	33
						49	-49	14
						-28	25	48
						-38	19	42
						-12	-56	11
						-9	-45	13
						-7	-37	36
						5	-35	38
						-12	-62	24
						-3	-74	31
						6	-68	30
						11	-54	35
						3	-62	41
	Binder et al., 2005b	24	Words	Read aloud	Concrete > Abstract	-60	-53	36
						-50	-58	32
						-49	-70	40
						60	-57	24
						48	-60	20
						62	-46	38
						43	-74	45
						61	-68	40
						47	-57	56
						44	-57	41
						44	-68	56
						56	-17	-22
						61	-51	4
						55	-6	-31
						52	-48	11

						70	-21	-16
						57	7	-37
						-28	54	-7
						-30	36	42
						-42	25	42
						42	27	39
						33	37	44
						41	37	32
						27	25	38
						21	48	17
						24	56	-8
						20	54	28
						24	39	31
						23	32	54
						13	53	-8
						-29	-39	-11
						-7	-52	37
						-7	-70	19
						-4	-70	42
						-16	-51	24
						0	-51	47
						9	-49	36
						10	-53	24
					Positive correlation imageal	43	-74	45
						61	-68	40
						47	-57	56
						44	-57	41
						44	-68	56
						56	-17	-22
						61	-51	4
						55	-6	-31
						52	-48	11
						70	-21	-16
						57	7	-37
						-28	54	-7
						-30	36	42
						-42	25	42
						42	27	39

						33	37	44
						41	37	32
						27	25	38
						21	48	17
						24	56	-8
						20	54	28
						24	39	31
						23	32	54
						13	53	-8
						-29	-39	-11
						-7	-52	37
						-7	-70	19
						-4	-70	42
						-16	-51	24
						0	-51	47
						9	-49	36
						10	-53	24
	Booth et al., 2006	13	Words	Semantic decision vs. phonological d	Semantic decision > phonol	-39	-57	18
	Demonet et al., 1992	9	Words	Lexical decision	Words > Phonemes	-48	-55	42
						-50	-55	38
						-50	-55	33
						-20	38	41
						-20	37	37
						-18	39	32
						-1	-60	28
						-1	-60	24
	Devlin et al., 2003	12	Words	Semantic decision vs. phonological d	Semantic decision > phonol	-10	52	-8
						-14	44	-8
						-20	30	48
						-42	-66	28
						-4	-56	28
	Fletcher et al., 1995	6	Sentences	Semantic decision	Story > unrelated Sent	-12	50	35
						-47	21	-24
						48	19	-25
						6	-65	15
						-46	-58	24
	Fliessbach et al., 2006	21	Words	Recognition	Concrete > Abstract	-36	-77	40
						-50	-65	39

						45	-69	39
	Gesierich et al., 2012	21	Faces	Same/different identity	Familiar faces > unfamiliar f	-3	-54	12
						-12	6	6
						-9	-6	6
						-3	-36	30
						-39	27	6
						18	21	-3
						-36	33	-12
						-39	15	-33
						-33	-72	39
						-60	-6	-18
						60	-3	-15
						-3	60	-9
						45	-66	30
						-21	57	0
						-54	-39	-6
						18	-45	-9
						36	-12	-18
						48	6	-27
	Gitelman et al., 2005	14	Words	Semantic decision vs. phonological d	Semantic decision > phonol	-15	-36	66
						-12	30	42
						-24	12	39
						-24	21	36
						-51	27	-3
						-48	15	9
						-54	27	24
						-9	15	60
						-39	-54	24
						-48	12	-15
						-42	0	-42
						-57	-45	3
						48	-15	-18
						51	-9	-9
						-51	-15	-9
						24	-72	-3
						15	-87	21
						-15	-45	-36
						6	-54	-33

						27	-78	-42
	Gourovitch et al., 2000	18	Words	Semantic generation vs. phonologica	Semantic generation > phon	-1	69	2
						-25	-15	-21
						-1	35	-17
						-8	35	-17
						-57	-24	-24
						47	-65	15
	Graves et al., 2010	20	Words	Reading aloud	Word frequency (positive)	1	-55	25
						-6	-29	40
						45	-63	39
						41	-29	27
						-36	-80	25
						-62	-41	31
						-30	22	43
	Homae et al., 2003	10	Sentences	Semantic decision vs. lexical decision	Story > unrelated sentences	-45	42	3
						51	36	-3
						48	36	15
						-24	18	48
						30	18	48
						-39	57	-6
						-51	-36	-6
						-54	-54	27
						51	-48	24
						36	-72	39
						-6	-63	39
	Jessen et al., 2000	14	Words	Memorise	Concrete > Abstract	-40	-68	43
						-44	47	2
						47	-61	48
						-1	-55	51
	Kosslyn et al., 1995	12	Picture and	Semantic decision	Category > Other	-52	-61	-7
						-28	-56	-8
						41	-74	24
						61	-58	-4
	Kotz et al., 2002	13	Words	Lexical decision	Related > unrelated	-46	-57	34
						-59	-34	39
						-17	-86	30
	Kuperberg et al., 2000	9	Sentences	Semantic decision	High meaning > Low meanin	-42	-44	-7
						-33	-45	10

						51	-25	1
						-45	-53	24
						39	13	-9
						-20	-82	-4
						-11	-53	23
						22	18	-21
						-2	-71	-5
						-12	-73	-12
						-6	19	-9
	Luo et al., 2002	12	Words	Semantic decision	Related > unrelated	-37	-78	40
						-39	-73	47
						43	-74	37
						-5	-53	10
						8	-53	9
						-21	-34	-14
						23	-31	-13
						-53	13	3
						-31	38	46
						-50	-70	26
	Mashal et al., 2007	15	Words	Semantic decision	Related > unrelated	-33	-69	47
						-41	-45	47
						44	58	10
						-38	51	15
						41	22	2
						61	-41	-3
						-55	-49	0
						-4	-10	3
						57	19	4
						-48	13	18
						-47	40	1
						-36	16	1
	Mechelli et al., 2007	20	Words/pic	Reading aloud and naming	Semantically related > unrel	-66	-38	-8
						-56	-24	-10
						-32	-72	44
						-58	-52	40
						2	30	40
						-6	18	44
						-46	24	-14

						-52	38	-6
	Mummery et al., 1998	10	Words	Semantic decision vs. phonological d	Semantic decision > phonol	-48	-72	35
						-59	-25	-8
						-31	-32	-19
						-47	-24	-19
						-5	66	18
						-14	58	-15
	Nichelli et al., 1995	9	Sentences	Semantic decision vs. orthographic d	Semantic decision > orthogr	-31	-58	47
						-22	25	43
						9	56	-19
	Price et al., 1997	6	Words	Semantic decision vs. phonological d	Semantic decision > phonol	-34	-5	-26
						-52	-69	21
	Raposo et al., 2006	15	Words	Semantic decision	Related > unrelated	44	25	30
						38	21	39
						44	13	31
						59	-41	2
						61	-33	-2
						55	-47	34
						6	37	37
						0	46	31
						-6	40	27
						51	33	9
						48	19	-8
						44	21	-1
						-42	23	26
						-36	13	23
						-44	23	36
						-48	-29	-5
						-63	-39	4
						-51	-12	-8
	Roskies et al., 2001	20	Words	Semantic decision vs. phonological d	Semantic decision > phonol	0	-27	-22
						-11	69	-9
						-13	55	24
						-23	26	36
						-26	3	29
						-26	-21	-2
						-37	26	-31
						-39	25	-20

						-40	-58	35
						-44	44	-17
						-52	-8	-19
						-5	43	-31
						-5	28	-32
						-54	23	-8
						-6	-58	17
						13	43	11
						17	-92	-25
						22	20	42
						41	-25	-25
						58	4	-15
	Sabsevitz et al., 2005	28	Words	Semantic decision	Concrete > Abstract	-28	-24	-24
						-26	-39	-14
						-61	-52	-14
						-48	-56	-15
						24	-5	-20
						28	-15	-24
						29	-28	-20
						-28	-79	44
						-32	-66	37
						-39	-72	49
						41	-59	36
						47	-69	36
						50	-44	46
						-23	28	-17
						-44	44	5
						-39	32	14
						-19	15	52
						-11	19	-20
						30	28	-16
						41	38	-18
						32	17	45
						50	36	7
						47	50	-4
						-14	-54	18
						8	-53	11
						3	-62	31

	Satpute et al., 2005	11	Words	Semantic decision	Causation > Association	-31	46	12
						-38	54	13
						28	-60	36
						-1	-24	-20
	Scott et al., 2003	9	Words	Semantic decision (living/non-living)	Semantics decision > syllabl	-3	48	42
						-49	34	-28
						-39	15	-38
						-59	-4	-23
						-36	-24	-25
						-63	-43	-3
						-53	-66	27
	Seghier et al., 2010	98	Words/pic	Semantic or perceptual decisions	Semantics > perceptual deci	-30	-66	42
						-48	-68	28
	Stringaris et al., 2008	11	Sentences	Semantic decision	High meaning > Low meanir	-54	-7	-11
						-22	-76	20
						52	20	15
						41	-1	42
	Sugiura et al., 2006	24	Words	Familiarity judgments	Personal names > famous n	-50	-70	41
						54	-72	37
						-9	-66	36
						12	-64	33
	Wallentin et al., 2005	18	Sentences	Semantic decision	Concrete > Abstract	-32	-44	-20
						30	-38	-24
						-16	-64	8
						14	-58	4
						42	-80	18
						-40	-82	16
						-4	-40	40
						-24	-74	38
						-32	28	44
						10	52	-12
	Whitney et al., 2009	18	Words	Semantic relatedness judgement	Positive correlation with nu	4	40	-20
						8	64	20
						-8	64	20
						0	52	-8
						-20	60	12
						-4	52	0
						-20	40	44

						-44	12	52
						-44	-72	36
						48	-72	36
						48	-72	28
						-60	-16	-24
						-8	-48	36
						-4	-68	36
						-40	8	28
						-36	20	28
						-8	16	48
						-4	12	52
	Wirth et al., 2011	21	Words	Living/non-living, syllable judgement	Semantic > phonological de	-48	17	-28
						-53	-20	-11
						-57	-68	21
						27	-86	-32
						-5	38	53
						-2	38	-11
						-2	-56	12
	Woodard et al., 2007	15	Words	Familiarity judgments	Familiar names > unfamiliar	-1	-51	22
						2	49	-8
						-18	36	38
						-49	25	-5
						27	26	38
						-57	-14	-17
						-46	-69	29
						-60	-41	-6
						53	-62	14
						29	-43	-22
						27	-21	-16
						16	8	16
						-10	-48	52
	Xu et al., 2005	22	Sentences	Memorise	Story > unrelated sentences	-44	34	-8
						52	30	-8
						-54	-10	-24
						56	-8	-28
						-58	-60	10
						60	-58	14
						-52	-72	28

**Bottom-up attention**	Arrington et al., 2000	12	Symbols	Posner cueing	Invalid > valid trials	52	-62	30
						51	-29	-9
						13	54	19
						3	23	54
						49	17	35
						46	25	-9
	Bledowksi et al., 2004	13	Shapes	Oddball	Relevant target > baseline	-56	0	24
						-38	5	8
						-48	-19	13
						-39	-25	53
						-51	-29	35
						-47	-40	46
						35	38	29
						7	-75	34
						55	-42	12
						39	7	9
						44	12	24
						36	36	30
						42	14	36
						43	-2	38
						2	12	40
						1	0	52
						44	-46	42
	Braver et al., 2001	14	Letters	oddball	Oddball	4	4	59
						0	0	46
						-36	-25	56
						-50	-4	3
						-56	-25	26
						-37	-77	-13
						35	-58	-47
	Cabeza et al., 2003	20	Letters	Blip detection	Visual attention > episodic r	-35	-25	58
						-43	9	-9
						-48	1	-4
						45	6	-6
						61	-2	-1
						-52	1	-4
						61	-56	-1
						-52	-75	12

						46	-78	18
						-15	-100	7
						42	-85	-2
						58	-30	23
						-67	-26	27
						34	-44	60
						29	56	14
						50	41	-7
						54	2	2
						55	13	44
						61	11	4
						10	2	58
						-63	8	11
						-7	-26	54
						-19	-41	64
	Chen et al., 2012	20	Spatial	Posner cueing	Invalid > valid	52	-42	15
						-26	-68	25
						30	-74	31
						-18	-68	59
						18	-62	55
						36	-48	53
						30	-6	53
						4	-56	53
						-50	-66	3
	Clark et al., 2000	6	Letters	Oddball	Oddball	0	-10	9
						0	-79	5
						-40	-64	-15
						32	-55	-15
						-64	-44	24
						-49	-19	57
						54	-36	41
						36	7	57
						-2	17	45
						-48	14	0
						-68	23	11
						49	20	-1
	Corbetta et al., 2000	13	Shapes	Posner cueing	Target > baseline	-25	-63	56
						-25	-55	48

						-28	-78	26
						38	-48	53
						31	-71	36
						59	-44	21
						9	-73	38
						-8	-70	47
	Doricchi et al., 2010	24	Symbols	Posner cueing	Invalid > valid > neutral	23	18	54
						45	10	43
						47	18	27
						66	-44	30
						15	-53	54
						39	-39	46
	Downar et al., 2001	10	Shapes and	Oddball	Relevant > irrelevant	64	-43	17
						-64	-48	20
						-2	29	35
						-38	22	3
						0	-55	45
						8	-13	8
						-49	-1	48
						-33	-10	68
						38	-67	-38
	Downar et al., 2002	10	Shapes, to	Oddball	Rare event > frequent event	62	-34	25
						61	-54	4
						47	4	19
						59	14	22
						48	16	-2
						-6	15	28
						-50	-50	-26
	Geng et al., 2009	15	Spatial	Posner cueing	Salient distractor > not > sal	-32	-44	45
						36	-43	43
	Gruber et al., 2009	12	Colour	Oddball	Oddball	-46	12	22
						-38	33	11
						62	33	5
						-37	-58	47
						41	-63	41
						-47	-56	-16
						10	23	47
						-24	19	48

	Hahn et al., 2007	22	Targets	Posner cueing	Uncued trials: positive corre	3	46	5
						2	-42	32
						-49	-65	37
	Indovina et al., 2007	12	Spatial	Posner cueing	Invalid > valid	-30	-58	44
						34	-68	38
						-42	6	32
						48	14	26
						-8	-74	42
						8	-60	52
						-32	24	-8
						34	26	-4
						8	20	40
						-6	18	46
						-30	4	54
						26	4	46
	Kiehl et al., 2001	10	Non-verba	Oddball	Relevant > irrelevant	31	59	-11
						29	57	-11
						-43	48	25
						26	9	73
						30	3	51
						-63	8	24
						2	3	57
						2	18	44
						10	32	20
						-51	5	-10
						54	5	-12
						-37	-9	64
						-35	-23	77
						67	-21	47
						-26	-39	78
						24	-44	78
						-54	-39	39
						55	-21	47
						-14	-65	69
						14	-47	78
						2	-31	38
						-60	9	-16
						61	20	-30

						62	-10	1
						-25	-4	-21
						20	0	-22
						-59	-17	15
						-57	-70	15
						2	-96	27
						-36	1	-10
						33	1	-16
						-7	-18	3
						17	3	12
						-40	-61	-26
						22	-56	-22
	Kincade et al., 2005	20	Spatial	Posner cueing	Invalid > valid	-60	-41	35
	Linden et al., 1999	5	Shapes or	Oddball	Relevant > irrelevant	58	-27	43
						-51	-19	50
						53	7	6
						-47	0	7
						1	5	46
						17	-100	11
	Macaluso and Patria, 2007	12	Spatial	Posner cueing	Invalid > valid	-30	-50	40
	Macaluso et al., 2002	8	Spatial		invalid > valid	71	-38	34
						-61	-51	33
						37	25	-28
						-49	25	-6
	Marois et al., 2000	20	Objects	oddball	Oddball	-21	-60	59
						-57	-33	25
	Mayer et al., 2006	17	Non-verba	Posner cueing	Invalid > valid	-55	7	25
						36	6	54
						45	15	30
						-36	19	-1
						60	-45	8
						-62	-45	14
						19	-67	42
						-3	-83	9
						-29	-66	-28
	Nardo et al., 2011	13	Object and	Saliency of stimulus in natural scene	Attention grabbing > not gr	58	-40	16
						4	-46	58
						22	2	56

						36	16	26
	Serences et al., 2005	12	Letters	Visual search	Relevant distractors > irrele	61	-43	25
						50	11	30
						30	4	47
						39	24	-2
						25	22	8
						18	-68	48
						25	-58	33
						6	17	47
						-58	-41	19
						-27	20	14
						-20	-58	47
						-26	-72	25
						-22	3	50
	Strange and Dolan, 2007	12	Words	oddball	Oddball	-48	-56	34
	Vossel et al., 2006	13	Spatial	Posner cueing	Invalid > valid	51	24	33
						60	-9	15
						63	-27	-9
						57	-57	15
						54	-45	45
						-42	-54	57
						33	3	-21
	Vossel et al., 2008	24	Shapes	Posner cueing	Invalid > valid and deviant >	66	-40	9
						-58	-54	13
						44	-62	13
						20	-72	59
						50	36	17
						36	20	-7
						-42	-36	53
						-50	-62	-15
						50	-58	-17
						-30	-36	-27
						54	12	41
						32	-66	51
	Vossel et al., 2012	26	Spatial	Posner cueing	Invalid > valid	59	-60	17
						-51	11	31
						34	5	44
						-29	-62	57

						39	-54	56
Episodic retrieval	Cabeza et al., 2003	20	Words	Remember, know, or new rating	Episodic retrieval > visual a	-20	70	1
						-48	46	-15
						-52	35	11
						-52	22	29
						-47	30	22
						-51	-54	48
						-43	-71	38
						-72	31	1
						-58	-42	-5
						-3	39	36
						2	-28	35
						-72	29	2
						-71	39	1
						-3	-92	25
						-15	-41	2
						-30	-12	69
	Cabeza et al., 2011	18	Words and	Episodic recall and perceptual target	Detection (memory and per	-62	-26	28
						46	-49	53
	Cansino et al., 2002	22	Pictures	Source localise or new	source correct > source inco	-20	51	44
						-3	40	61
						-40	7	16
						58	1	55
						-50	3	57
						-39	-3	64
						2	-30	68
						-46	-40	36
						-61	-56	6
						-44	-54	4
						55	-73	2
						31	-78	-9
						42	-49	-14
						-40	-59	-25
						-14	17	-19
						-10	-60	-19
	Ciaramelli et al., 2010	14	Words	Old/new cues or no-cues	Uncued > cued	-40	-66	24
						20	-32	60
						-46	10	26

						-42	44	6
						-26	10	56
						60	-12	12
						-32	-80	32
						-34	-66	26
	Daselaar et al., 2006	14	Words	Old/new (confidence ratings)	Recollection correlation	-26	-26	-11
						30	-23	-11
						-4	-43	20
						0	-23	33
						-45	-65	31
						-27	14	-20
						34	17	-20
						0	-81	-2
						-4	59	-3
					Definitely old > definitely ne	-26	-26	-11
						30	-23	-11
						-4	-43	20
						0	-23	33
						-45	-65	31
						-27	14	-20
						34	17	-20
						0	-81	-2
						-4	59	-3
	Eldridge et al., 2000	12	Words	Old/new (remember, know if old)	Remember > know	-31	40	42
						16	-18	47
						57	-4	40
						-45	-54	46
						59	-56	39
						61	12	21
						-52	-40	28
						-46	-64	25
						20	-58	18
						-61	-39	12
						-12	19	-1
						-38	-7	-3
						-34	-24	-13
						24	-40	-12
						38	-58	-16

	Fan et al., 2003	8	Pictures ar	Source localise or new	Source > new	27	-85	-19
						-36	58	-4
						4	52	31
						-33	-48	43
						-50	25	17
						-47	21	-10
	Fenker et al., 2005	20	Faces and	Remember, know, new	Remember > know	15	12	-21
						-27	12	-21
						-9	42	54
						-39	9	60
						-33	33	12
						-9	54	-9
						63	-9	-21
						-57	-54	0
						66	-42	-12
						51	-66	21
						33	-27	9
						-54	-15	-15
						-66	-30	-12
						-57	-39	12
						54	3	9
						42	-6	42
						69	-18	21
						-39	-69	36
						-39	-72	51
						6	-66	21
						27	-93	21
						36	-81	24
						-24	-78	3
						-33	-93	3
						-36	-81	15
						6	-93	27
						-6	-96	21
						18	-87	-24
						-21	-96	-6
						-12	-6	-18
						33	-18	-12
						33	6	-30

						-33	-27	-15
						-36	-18	-24
						0	-39	39
						24	-72	-39
	Henson et al. 1999	12	Words and	Remember, know, new	Remember > know	-51	42	-21
						-57	30	30
						-64	32	17
						-12	70	9
						-5	46	21
						-34	-58	52
						-53	-43	44
						-12	-37	3
						-5	-21	28
						-5	-40	40
						-5	-74	50
						2	-65	39
	Henson et al., 2005	22	Words	Old, new	Deep learning > shallow lea	-2	67	12
						1	55	-13
						-5	55	47
						18	51	47
						59	46	-14
						-45	28	-27
						-51	31	7
						53	29	-22
						-45	8	-38
						-38	20	-43
						46	7	-46
						-35	-6	-47
						-32	2	-28
						-22	-8	-37
						-15	-14	-27
						-32	-33	-28
						30	-36	-25
						-64	-38	-10
						-5	-50	34
						-50	-63	33
						15	-105	26
	Herron et al., 2004	12	Words	Old, new	Old > new	-11	-65	46

						-34	-72	37
						11	-38	19
						11	-62	38
						41	-65	41
	Hutchinson et al., 2012	26	Words	Episodic recall and specificity of reca	Recollection detail (Specific	-33	15	51
						57	27	30
						57	21	36
						57	21	36
						60	15	27
						-3	33	-15
						33	36	-9
						-9	54	42
						0	36	42
						-12	39	48
						-36	-39	42
						-45	-33	39
						-42	-42	57
						-3	-57	27
						-3	-69	36
						-3	-39	6
						-15	-69	27
						42	-69	39
						36	-66	57
						36	-57	42
						-39	-72	42
						-42	-66	54
						-39	-63	39
	Iidaka et al., 2006	32	Pictures	Old, new	Correct > CR X semantic > sp	-32	-50	38
						-56	16	22
	Johnson and Rugg, 2007	26	Words ove	Remember, know, new	Remember > know	-21	-24	-21
						-42	-78	30
						-3	60	0
						-42	-33	-21
						-54	-57	-21
						-36	-42	-30
						15	-27	69
						33	-24	57
						51	-9	45

						-24	-6	21
						21	-3	24
						-15	-39	60
						3	-51	21
						-9	57	-9
						-6	-9	66
						54	-72	18
						57	-3	-18
						-27	-60	15
						0	-24	15
						33	-93	-3
						45	-78	12
						57	-60	-18
						0	-66	-27
						36	-81	33
	Jonides et al., 1998	12	Non-words	Old/new	Old > new	41	58	-15
						-54	34	25
						47	26	17
						0	16	36
						0	16	36
						44	-48	48
						27	-94	-6
						-25	-96	-3
						-46	-72	24
						60	3	-24
						-56	-9	-23
						20	-53	20
						57	-63	13
	Kahn et al., 2004	17	Words	Source or new	Source correct > source inco	36	-48	42
	Kao et al., 2005	20	Pictures	Old, new	Remember > forgotten	-22	-72	45
						38	-60	-15
						45	6	22
						-45	-66	-4
						38	-84	15
						-34	30	-22
						-41	6	26
						15	-54	15
						34	-48	49

	Kensinger et al., 2006	17	Words and	Old/new	Source correct > source inco	-33	1	64
						-34	50	-15
						-44	55	-23
						-28	43	-16
						-50	-24	56
						-36	-38	67
						-50	-44	50
						-36	-34	58
						-25	-11	-20
						24	12	6
	Kim and Cabeza, 2007	16	Words	Old/new (confidence ratings)	High confidence > low confi	27	-27	-8
						-27	-27	-11
						27	3	-16
						-8	33	-5
						15	33	6
						15	-54	30
						4	10	-10
						-46	-80	25
						23	-38	65
	Leveroni et al., 2000	11	Faces	Old, new	Newly learned > new	-5	14	59
						-38	-67	48
						-4	-48	50
	Lundstrom et al., 2003	21	Picture-wo	Source memory and old/new	Source memory > new	-5	-64	35
						-5	-69	43
						-50	-49	50
						39	-69	46
						-44	45	5
						-40	57	-5
						-40	40	21
						-47	53	0
						-49	33	15
						-44	21	0
						-27	61	10
	McDermott et al., 2000	24	Words	Old, new	Old > new	46	-54	54
						-38	-49	41
						40	-53	47
						50	29	25
						-62	-61	29

						52	-47	33
						-48	41	1
	Montaldi et al., 2006	13	Pictures	Old/new/high fam/low fam	Recognition > high familiarit	-48	27	-15
						42	24	-21
						-3	48	12
						-42	-72	30
						-12	-33	-3
						21	-33	-3
	Moritz et al., 2006	17	Words	Old/new (confidence ratings)	High confidence > low confi	-6	36	-6
						-3	-18	36
						-15	-51	-9
						3	-87	27
						-27	-33	-12
						-51	-66	39
						-36	0	15
	O'Connor et al., 2010	19	Words	Old/new valid or invalid cues	Invalid > valid cued	-27	18	-9
						-30	60	3
						-39	6	36
						-12	36	60
						-48	36	24
						-45	12	54
						27	60	18
						-42	-84	-9
						39	-87	-12
						-30	-96	12
						42	-81	6
						-42	-54	42
						-21	-78	42
						42	-54	39
						63	-45	-6
						51	-51	6
						21	-15	-12
						-12	-3	18
						15	6	-3
						-6	-30	27
						12	33	24
	Otten and Rugg, 2001	15	Words	Old, new	Subsequently forgotten > su	2	-14	40
						-38	46	25

						43	47	27
						-57	-52	42
						70	-43	29
						-1	-75	43
	Shannon and Buckner, 2004	72	Pictures ar	Old, new	Old > new	0	-72	36
						-46	-59	49
						-5	-56	30
	Tsukiura et al., 2005	22	Words (Ch	Old, new	Old > new	-11	-52	46
						-53	-61	39
						-56	-34	43
						-16	-35	-10
						10	-38	-3
	Vilberg and Rugg 2009	20	Pictures of	Old/new (remember, know if old)	Remember > know	-36	30	-21
						-27	27	54
						39	-21	57
						21	-15	-36
						-24	-24	-30
						24	-27	-30
						-63	-51	-12
						-6	-54	18
						-51	-75	21
	Vilberg and Rugg., 2007	23	Pairs of pic	Remember, know, new	Remember > know	-9	-78	33
						-18	33	51
						-48	27	33
						-36	21	51
						36	-27	69
						6	9	-3
						-15	-3	-15
						-36	-9	-36
						-51	-57	-21
						-24	-87	-21
						30	-33	-21
						33	-60	57
						-36	-78	33
	Vilberg and Rugg., 2012	21	Words/pic	Old/new, maintain over a delay	Old > new and sustained > t	-48	32	13
						-51	26	28
						6	35	46
						9	11	1

						9	-7	-8
						33	23	-5
						-57	-46	-14
						63	-37	-20
						-33	-55	46
						-39	-55	55
						-30	-73	46
						42	-55	43
						39	-70	34
						12	-82	-29
						27	-67	-35
	Weis et al., 2004	16	Pictures	Old/new (confidence rating)	Positive recognition effect	45	-59	36
						1	-64	-21
						-16	-52	-26
						19	-5	58
						-21	-78	-28
	Wheeler and Buckner, 2004	25	Words	Remember, know, or new rating	Remember > know	-52	-54	38
						-43	-71	40
	Woodruff et al., 2005	17	Words and	Remember, know, new	Old > new	-12	26	58
						-44	14	42
						-30	14	46
						-2	-16	42
						-60	-24	-10
						-64	-46	6
						-50	-56	26
						-4	-72	38
						-42	-74	-10
						10	-8	0
						4	-26	68
						-4	-42	32
						66	-6	-22
						58	-24	-16
						52	-52	44
	Yonelinas et al., 2005	16	Words	Old/new (confidence ratings)	Recollection correlation	-6	54	-6
						9	63	21
						-30	39	-12
						24	30	-15
						63	3	27

						-3	-54	6
						0	-45	30
						60	-24	15
						-54	-27	18
						-21	-42	63
						36	-36	60
						-45	-21	39
						54	-12	48
						-42	-54	15
						-54	-42	21
						-45	-3	-3
						54	-69	9
						57	-48	3
						-54	-63	0
						66	-39	-3
						-24	-21	-21
						30	-21	-21
						-15	-51	-15
						15	-60	-15
						-45	-6	12
						18	-3	12
Numerical calculation	Chochon et al., 1999	8	Numerals	Multiplication	Multiplication > digit namin	-54	9	39
						-31	-72	40
						-47	-33	40
						54	-25	51
	Dehaene et al., 1999	7	Numerals	Addition	Exact > approximate (i.e. Ve	-34	70	-6
						-8	67	7
						-7	-56	23
						23	-81	34
						45	-77	25
						-46	-72	44
						53	-15	5
	Delazer et al., 2003	13	Numerals	Multiplication	Untrained multiplication > r	-7	-75	52
						-49	20	-15
						-53	12	18
						-26	10	62
						-1	-80	17
						-5	-88	-23

						19	-67	69
						55	21	19
						33	24	-21
						49	38	13
						6	29	33
						34	-99	-9
						44	-78	-33
	Delazer et al., 2004	13	Numerals	Multiplication	Multiplication untrained > t	-20	-52	64
						-68	-22	26
						-53	10	18
						-66	-35	22
						-7	-88	4
	de Pisapia et al., 2007	20	Numerals	Mental arithmetic	Mental arithmetic	-5	11	65
						21	26	19
						-31	-36	40
						-64	-6	17
						40	-64	-19
						-38	-83	-13
						-25	-91	2
						-31	-69	33
						14	-92	-6
	Fehr et al., 2007	11	Numerals	Multiplication	Complex > simple	4	25	45
						-40	49	33
						-42	38	29
						49	33	20
						49	16	25
						31	-69	-15
						-7	-36	-1
						-3	-5	5
						-3	-20	9
	Grabner et al., 2007	25	Numerals	Multiplication	Multi-digit > single-digit	36	-68	-26
						-28	26	-4
						30	30	-2
						-36	-42	44
						-46	8	30
						-42	-66	-30
						0	-74	-24
						10	26	36

						-26	-2	54
						-10	16	44
						0	-70	10
						38	-46	42
						24	-28	6
	Hanakawa et al., 2003	16	Numerals	Addition	Mental operation > verbal r	-38	-62	-28
						30	-58	40
						-24	-68	40
						30	-52	34
						34	2	54
						-30	-8	54
						-50	8	22
						-44	28	16
						38	-74	20
	Ischebeck et al., 2006	12	Numerals	Multiplication	Untrained > trained	-3	9	51
						-51	6	-27
						-18	-21	15
						42	-45	45
						-33	-66	39
	Ischebeck et al., 2007	18	Numerals	Multiplication	Novel > repeated	-6	15	39
						15	6	12
	Ischebeck et al., 2009	17	Numerals	Multiplication	Untrained > trained	-42	9	24
						-24	18	3
						-6	-15	51
						-30	6	57
						30	30	6
						45	39	15
						30	9	57
						-18	-3	18
						18	3	21
						24	-30	6
						-24	-57	51
						48	-33	51
						24	-63	63
						18	-69	51
						-21	-87	3
						-30	-69	30
	Kong et al., 2005	16	Numerals	Arithmetic	Complex > simple	-3	15	51

						6	21	42
						-45	9	27
						-24	-78	36
	Krueger et al., 2008	18	Numerals	Integrate/ font verification	Integration > font verificatio	-46	-54	50
						-42	21	42
						-29	22	51
						-51	27	25
						-6	-68	68
						-37	-69	57
						-7	-71	44
						45	-71	43
						-33	-79	50
						2	-30	38
						-3	-37	22
						-3	-28	25
	Lee et al., 2000	11	Numerals	Arithmetic	Multiplication > subtraction	-52	-54	36
						-23	41	41
						13	-78	8
						57	-5	22
						3	56	8
						-1	-41	54
	Menon et al., 2000	16	Numerals	Arithmetic	3-operand > digit strings	34	-75	47
						-26	-77	51
						-50	-47	57
						-53	-66	-8
						-38	-73	-37
						2	25	58
						-46	15	24
						-45	29	-11
						-37	4	45
						57	17	17
						36	29	-6
						40	-69	-46
						32	12	65
						-16	-2	3
						-12	-13	6
	Menon et al., 2002	16	Numerals	Arithmetic	Incorrect > correct	-48	27	20
						-49	45	-15

	Molko et al., 2003	14	Numerals	Arithmetic	Number size effect; large > s	-46	-49	59
						32	-67	44
						-12	-83	-32
						2	28	46
						28	20	56
						15	-71	47
						-34	-75	-37
	Prabhakaran et al., 2001	7	Sentences	Arithmetic	One operation > no operati	-45	60	-5
						-47	57	4
						-53	32	16
						-42	37	19
						56	36	18
						-43	23	30
						43	39	27
						-48	22	39
						40	19	38
						36	44	-14
						2	67	16
						-35	21	44
						-3	31	43
						-43	8	31
						46	17	29
						15	-44	26
						5	23	38
						61	-91	-1
						-66	-43	5
						-57	-43	-4
						35	-56	36
						-46	-42	45
						-45	-42	51
						43	-39	49
						-36	-56	58
						24	-60	57
	Rickard et al., 2000	8	Numerals	Multiplication	Multiplication verification	-25	-58	49
						33	-59	50
						-43	-58	-12
						35	-81	-8
						-42	18	30

						50	21	17
						-36	55	15
						45	55	14
	Sammer et al., 2007	20	Numerals	Arithmetic	Addition > reference	-33	-55	57
						45	-39	46
						-18	7	58
						-31	15	24
						-12	-8	10
						39	-60	52
						38	42	15
						-5	17	48
	Simon et al., 2002	10	Numerals	Arithmetic	Subtraction > letter control	-7	15	52
						6	41	50
						-28	-1	63
						-37	-55	33
						-28	7	58
						-46	38	24
						-47	62	4
						-59	17	26
						49	25	19
						57	11	7
						-7	30	24
						6	30	28
						-34	31	-7
						40	27	-8
						-50	2	54
						-33	-70	61
						36	-58	50
						-11	-70	56
						-63	-28	49
						-37	-63	43
						-29	14	-5
						18	11	3
						-42	18	-6
	Stanescu-Cosson et al., 2000	7	Numerals	Arithmetic	Calculation > letter matchin	-55	17	22
						-50	23	48
						36	-62	50
						54	-32	51

						36	-59	32
						53	51	16
						45	23	37
						48	35	-13
						-41	-53	55
						-63	-41	54
						-55	48	-8
						-64	45	10
						-55	51	23
						-7	41	50
						-3	33	55
						6	-79	12
	Wood et al., 2008	17	Numerals	Arithmetic	Multiplication > non-multip	-52	-60	30
						-15	56	30
	Zago et al., 2008	14	Numerals	Addition	Number manipulation > ma	10	36	28
						-26	8	54
						-46	10	22
						-48	44	18
						-30	30	-6
						36	4	54
						54	14	10
						48	52	20
						38	26	-2
						12	8	0
						-12	10	4
						-48	-38	42
						-54	-34	46
						-26	-66	46
						32	-64	48
						44	-42	42
						-52	-56	-14
						2	-74	-16
**Numerical fact retrieval**	Ansari et al., 2007	13	Dot arrays	Smaller/Larger judgement	Small array > Large array	-41	-33	51
						34	-42	50
	Chochon et al., 1999	8	Numerals	Multiplication	Multiplication > digit namin	-54	9	39
						-31	-72	40
						-47	-33	40
						54	-25	51

	Dehaene et al., 1999	7	Numerals	Addition	Exact > approximate	-34	70	-6
						-8	67	7
						-7	-56	23
						23	-81	34
						45	-77	25
						-46	-72	44
						53	-15	5
	Delazer et al., 2003	13	Numerals	Multiplication	Trained > untrained	-48	-60	29
						-49	-15	-38
						-11	-43	27
						-22	-42	36
						-24	-15	51
						-23	-64	-34
	Delazer et al., 2004	13	Numerals	Multiplication	Trained > untrained	-7	-88	4
						-48	-60	29
						-49	-15	-38
						-11	-43	27
						-22	-42	36
						-24	-15	51
						-23	-64	-34
	Grabner et al., 2007	25	Numerals	Multiplication	Single digit > multi-digit	-58	-64	32
	Grabner et al., 2009a	28	Numerals	Arithmetic	Retrieval > calculation	-57	-65	43
	Grabner et al., 2009b	28	Numerals a	Multiplication and figural-spatial cal	Trained > untrained	-60	-48	39
						57	-57	24
						-3	-42	36
	Grabner et al., 2011	34	Numerals	Arithmetic verification	Confusion > non-confusion	-51	-51	36
						-21	42	30
	Ischebeck et al., 2006	12	Numerals	Multiplication	Trained > untrained	-48	-66	39
	Ischebeck et al., 2007	18	Numerals	Multiplication	Repeated > novel	-66	-30	-6
						66	-39	27
						-51	-63	33
	Ischebeck et al., 2009	17	Numerals	Multiplication	Trained > untrained	-45	-69	45
						-9	-48	33
	Lee et al., 2000	11	Numerals	Arithmetic	Multiplication > subtraction	-52	-54	36
						-23	41	41
						13	-78	8
						57	-5	22
						3	56	8

						-1	-41	54
	Rickard et al., 2000	8	Numerals	Multiplication	Multiplication verification	-25	-58	49
						33	-59	50
						-43	-58	-12
						35	-81	-8
						-42	18	30
						50	21	17
						-36	55	15
						45	55	14
	Rosenberg et al., 2011	20	Numerals	Mathematical verification	Identification > calculation	50	-66	14
						-48	-72	38
						-50	-58	20
						6	-60	60
						6	-68	60
						48	-54	34
						48	-64	16
	Stanescu-Cosson, et al., 2000	7	Numerals	Arithmetic	Exact > approximate	-34	70	-6
						-42	65	-15
						-8	67	7
						-7	-56	23
						23	-81	34
						45	-77	25
						-46	-72	44
						-46	-64	29
						-64	-70	-28
						53	-15	5
						62	-10	4
						75	-11	-5
	Wood et al., 2008	17	Numerals	Arithmetic	Multiplication > non-multip	-52	-60	30
						-15	56	30
	Wu et al., 2009	18	Arabic and	Verification	Arabic > Roman	-46	-72	20
						-4	56	24
**Phonological decision**	Baciu et al., 2005	10	Words	Rhyme or perceptual judgement	Rhyme > visual detection	-48	-59	-7
						-48	-47	-12
						-42	31	2
						-51	13	21
						-24	-60	36
						-24	-58	44

						-50	-33	20
						-44	45	16
						-36	-71	32
	Bohland et al., 2006	13	Syllable se	Go/NoGo overt reading	Complex syllable > simple sy	0	18	46
						0	4	62
						0	0	70
						4	24	38
						50	22	-6
						42	20	-12
						38	26	0
						38	24	-6
						-26	-62	52
						-30	-54	52
						-48	-40	52
						-20	-66	66
						-38	-44	44
						-34	26	0
						-34	22	4
						-50	12	0
						22	-76	-20
						26	-62	-18
						0	16	48
						-8	8	62
						2	34	36
						8	26	34
						0	16	66
						2	14	32
						-6	24	28
						34	22	-8
						38	44	24
						52	20	-4
						40	20	10
						52	34	26
						58	24	14
						-42	30	24
						-30	24	6
						-42	46	22
						-36	16	-8

						-58	14	18
						-52	16	14
						-44	14	4
						-62	6	28
						-40	12	26
						-52	10	44
						-50	4	36
						42	-50	-30
						28	-52	-24
						32	-52	-28
						36	-56	-28
						-2	-72	-8
						14	-66	-12
						42	-72	-28
						2	-56	-32
						14	-58	-20
						14	-54	-14
						16	-6	14
						-10	0	10
						10	-2	12
						-4	-10	14
						8	-8	2
						-30	-52	50
						-40	-44	54
						-52	-40	56
						-36	-48	42
						-24	-72	46
						-18	-68	64
						34	2	58
						34	2	38
						34	4	44
						44	12	38
						34	0	48
						-44	-58	-16
						-32	0	52
						-38	0	62
						-38	-4	42
	Booth et al., 2002	13	Visual and	Rhyme judgement / Semantic associ	Rhyming > Meaning	-6	-36	45

						-57	-21	39
						0	-39	24
						-6	-63	24
						-9	57	-12
					Rhyme > spell	-3	-9	48
						63	-24	24
						48	-39	24
						-57	-42	24
						-63	-30	15
						39	-30	15
						54	-6	3
						-42	6	-6
						-48	-15	51
						-6	-24	45
						-3	-72	36
						-42	-69	33
						63	-21	27
						-57	-21	21
						-42	6	-9
						0	57	-12
	Booth et al., 2006	13	Words	Phonological > Semantic	Phonological > Semantic	-36	42	-21
						-18	-66	54
						-54	-27	51
						-24	-3	48
	Cousin et al., 2007	11	written wo	living or nonliving? / rhyme judgeme	Phonological > Semantic	-53	5	3
						-47	50	36
	Demonet et al., 1994	9	Non-words	Phoneme detection	Phoneme > tones	-59	-1	-3
						-29	-72	-6
						-44	-49	-12
						-52	-11	47
						-7	-63	15
	Desai et al., 2006	25	Words	Verb generation	Irregular > regular verbs	9	5	1
						-33	-54	45
						-51	9	21
						36	-62	45
						39	27	-1
						51	7	31
						-30	-75	26

						-45	-56	-4
						-51	33	-1
						-47	-1	41
						-12	-27	6
	Devlin et al., 2003	12	Words	Phonological > Semantic	Phonological > Semantic	-44	38	-20
						-50	6	24
						44	36	26
						46	28	-16
						36	24	-8
						-30	20	-8
						-42	34	12
						6	36	42
						24	52	-8
						46	-44	44
						-42	-40	46
						28	-68	44
						-20	-68	46
						-48	-66	-20
						52	-56	-16
	Fiebach et al., 2007	12	written wo	Item present or not	Phonological > Semantic (re	46	-3	-5
						49	-66	27
						12	54	-7
	Ghosh et al., 2008	10	Syllables	Syllable reading	Bisyllable > monosyllable	-52	-6	30
						-46	-8	54
						50	-4	38
						-56	16	20
						-60	16	32
						-6	4	68
						8	16	44
						54	-2	32
						-24	-70	42
						-20	-70	48
						24	-68	50
						-32	-52	50
						-62	-2	-10
						-66	-18	2
						70	-2	-6
						70	-28	4

						-58	6	-12
						-42	-64	-10
						18	-92	-12
						-38	-50	-24
						-40	-44	-24
						-22	4	4
						26	4	0
						22	8	4
						-22	0	-4
						-30	-84	-20
						-8	-80	-32
						-30	-60	-28
						-32	-56	-26
						28	-62	-26
						2	-102	-6
						14	-66	10
						-46	-84	-2
						-28	-82	16
						-16	-92	-10
						34	-88	20
						34	-80	-16
	Gitelman et al., 2005	14	Words	Phonological > Semantic	Phonological > Semantic	15	-81	-36
						-57	9	24
						-48	3	18
						42	33	-12
						51	39	0
						51	45	-12
						-45	-3	48
						42	12	-18
						27	9	-21
						18	-15	-21
	Gold and Buckner., 2002	24	written wo	Short or long vowel item	Phonological > Semantic	-43	-41	38
						-58	3	27
	Gold et al., 2005	32	written wo	Regularise irregular words	Phonological > Semantic	-43	-41	38
						-58	3	27
	Gourovitch et al., 2000	18	Words	Phonological > Semantic	Phonological > Semantic	-37	-77	26
						25	38	23
						46	13	2

						-57	5	18
						-38	8	-5
						48	7	-15
	Gruber et al., 2010	18	Letter sequ	Phonological rehearsal or orthograp	Phonological > orthographi	-36	52	12
						36	44	28
						-44	-40	40
						48	-40	40
						0	12	52
						-28	-60	40
						32	-56	44
						-40	16	-4
						32	24	-4
						-36	-56	-36
						24	-64	-28
						-16	-4	16
						20	4	16
						-44	8	24
						-48	-56	-16
						-4	-24	-16
	Heim et al., 2008	28	written wo	Phonological Fluency	Phonological > Semantic flu	-50	10	21
						-40	-40	47
	Katzir et al., 2005	12	Objects	Phonological judgement/ perceptua	Phonological blend > percep	-39	-69	-12
						-51	15	33
						33	-93	-3
						33	30	-12
						-3	36	45
						-6	-21	-6
						30	-33	0
						-27	-60	42
						12	3	0
						45	24	27
						42	3	27
						33	-93	-3
						12	18	45
						-33	-84	-15
						-45	21	24
						33	21	0
						-30	-60	45

						-36	-12	-33
						27	-33	-3
						21	-6	15
						0	-45	-21
						-60	-45	-3
						51	36	30
	Kuo et al., 2004	10	Chinese ch	Homophone or orthographic judgem	Homophone > orthography	-48	0	46
						-4	14	52
						-44	10	34
						-50	24	26
						-46	18	18
						-42	6	18
						-24	-62	44
						-38	-46	52
						-54	-42	14
						-34	-84	4
						-42	-62	2
						-32	-78	-2
						-36	-54	-8
						8	28	36
						42	14	26
						32	-84	6
						-46	2	44
						-4	14	56
						-40	14	30
						-48	26	22
						-46	16	22
						-46	4	22
						-48	16	2
						-42	-54	42
						-56	-44	16
						-34	-72	8
						-30	-78	-4
						36	-62	-10
						28	-70	-14
	Liu et al., 2008	16	written & s	Semantic association /rhyme judger	Phonological > Semantic	-48	2	24
						-36	-40	28
						-38	38	16

	Lurito et al., 2000	5	Words	Rhyme judgement	Phonology > orthography	-48	38	21
						-52	16	31
						-58	16	3
						-67	-29	29
						-60	-48	2
						-47	-63	-18
						61	10	0
	McDermott et al., 2003	20	written wo	Rhyme judgement / Semantic associ	Phonology > Semantic	-58	6	13
						48	-35	49
						-32	-54	55
						35	-44	56
						48	-63	-6
	Mummery et al., 1998	10	Words	Phonological > Semantic	Phonological > Semantic	35	19	-25
						31	10	-31
						-5	-57	15
						-5	15	7
	Paulescu et al., 1997	5	written wo	Phonological Fluency	Phonological > Semantic flu	-38	10	18
	Price et al., 1997	6	Words	Phonological > Semantic	Phonological > Semantic	-3	9	4
						-42	-44	36
						38	-48	40
						45	-30	42
						32	-61	37
						-55	2	23
						-13	-82	26
	Roskies et al., 2001	20	Words	Phonological > Semantic	Phonological > Semantic	9	-72	-27
						-26	28	-2
						-2	-8	3
						-37	-76	-19
						-39	-1	5
						-52	3	25
						-52	6	14
						-58	-7	40
						18	-6	14
						32	-72	-20
						34	-68	-31
						7	-70	4
	Scott, Leff and Wise 2003	9	written wo	Three syllables or not?	Phonological decision > Hur	-60	-26	39
						-48	-51	60

						-20	-67	58
						25	7	48
						49	-48	55
						11	-73	62
						30	-59	41
	Seghier et al., 2004	30	written wo	Rhyme detection > Semantic categor	Phonology > Semantics	-40	-54	19
						-27	51	27
						-54	-35	40
						-22	-77	55
						-7	5	58
	Simon et al., 2002	10	Words	Phonological detection	Phonology > orthography	-4	12	56
						-56	20	32
						-44	16	12
						52	16	8
						-52	0	48
						-64	-36	36
						-28	-80	40
	Simon et al., 2004	10	Words/nu	Phoneme detection/number calcula	Phoneme and calculation co	-48	20	0
						-58	6	20
						-42	26	20
						-50	18	28
						-46	9	44
						-2	-26	44
						-28	-72	44
						50	21	4
						6	29	48
	Specht et al., 2003	15	Words, pse	Pseudo-word/tone detection task	Pseudo-word > tones	-64	-24	4
						60	-12	0
						4	40	40
						36	24	-8
						-28	20	-4
						-8	36	28
	Tyler et al., 2005	18	Words	Phonological similarity judgment	Regular > irregular	-46	-26	8
						-56	-24	10
						-56	-48	8
						58	-14	2
						58	-28	4
						62	-6	10

						46	-42	34
						38	-44	42
						-50	12	20
	Wildgruber et al., 2005	10	Sentences	Vowel identification/emotion identi	Vowel identification > emot	-54	6	33
						-48	-39	54
						27	-66	39
	Xu et al., 2001	12	Pseudo-wo	Rhyming or colour task	Pseudo-words > colour task	32	-68	-20
						-48	-54	-14
						-44	4	28
						-34	-50	38
						10	-72	-46
						-46	-66	-10
						32	-76	-14
						-52	10	12
						-50	-4	46
						-38	-48	50
	Xu et al., 2002	18	Words	Rhyme judgement	Phonology > orthography	-38	46	24
						-4	4	62
						-54	28	24
						-38	22	16
						-52	14	20
						-52	4	44
						-52	-44	24
						-2	10	58
						-54	30	24
						-54	14	20
						-42	-4	54
						-48	6	42
						-56	12	-6
						-56	-44	28
						-18	52	14
						32	46	22
						-46	18	30
						-38	28	-2
						-32	-70	38
						-44	-40	30
						42	52	20
						4	36	52

						-46	12	28
						-34	-64	46
	Zahn et al., 2004	14	Words and	Word discrimination	Phonological and lexical cor	-40	20	28
						-36	-64	42
						-52	-16	-14
						-56	-8	28
						44	-8	42
						8	-76	0
**Sentence-level processing**	Bottini et al., 1994	6	written ser	Sentence plausible or not	Metaphor > literal meaning	38	11	47
						44	32	0
						-1	47	0
						62	-39	-2
						21	-38	30
						21	-62	24
	Boulenger et al., 2009	18	written ser	Sentence verification. Does a subsec	Idioms > Literal meaning	-48	28	6
						-58	16	10
						-44	30	2
						-56	20	26
						-62	-56	6
						20	-82	-32
	Caplan et al., 2003	13	Sentences	Plausibility judgement	Object relative clause > Sub	-2	-53	55
						10	23	47
	Chen, Widick and Chatterjee 2008	14	written ser	Sentence plausible or not	Predicate metaphor > litera	-56	21	2
						-53	6	-33
						-56	-10	-19
						-66	-38	8
						-44	-56	39
						58	-2	-33
	Collette et al., 2001	12	written ser	Sentence completion	Inappropriate > appropriate	-33	21	27
						-40	48	-13
						-55	24	14
						-46	25	18
	Constable et al., 2004	20	Sentences	Plausibility judgement	Object > subject relative cla	-65	-50	8
						-63	-33	3
						-48	38	-4
						-60	-53	22
						-5	-51	38
	de Diego Balaguer et al., 2006	12	Non-words	Generate the present tense	Nonword inflection > repeti	-48	16	12

						-12	-52	-32
						28	-24	56
	Eviatar and Just 2006	16	written ser	stories followed by yes no comprehe	Metaphor > Literal and Iron	-45	21	15
						-52	-53	9
						-26	-77	8
						48	-61	3
	Fiebach et al., 2005	14	Sentences	Probe questions	Long prepositional phrase >	-20	-52	26
						22	-59	23
						-13	-63	50
	Grossman et al., 2002	24	Sentences	Who is the agent?	Object > subject relatives)	-47	19	-1
						-7	-71	47
						35	22	-21
						62	-43	21
	Haller et al., 2007	20	Sentences	Is meaning reversed?	Complex > medium	-2	-67	9
						-42	10	43
						0	11	59
						-55	-39	13
						35	1	59
						47	21	33
						-32	29	9
						-58	-3	-5
						36	32	7
						34	-49	43
	Hoenig and Scheef 2009	22	written ser	Context verification /semantic relate	Ambiguous > unambiguous	-26	8	-26
						-6	46	38
						-46	-62	38
						-14	20	54
						-8	-64	22
						-36	10	44
						20	38	44
						54	-64	40
						28	-14	-34
						-2	42	-6
						32	22	-26
						-44	10	-40
						-6	48	8
						-54	-60	36
						-34	10	46

	Homae et al., 2002	9	Sentences,	Probe detection	Auditory Phrases > nonword	-57	27	6
						57	21	9
						-45	39	24
						-39	60	-9
						45	51	-9
						-27	18	51
						30	18	48
						48	15	36
						-30	15	12
						45	-3	9
						-60	-12	-12
						57	-18	-9
						-54	-36	-3
						60	-39	-12
						-48	-69	21
						-45	-54	54
						48	-69	33
						63	-21	21
						6	-18	48
						-3	-36	36
						-9	-81	33
						-3	-72	24
	Humphries et al., 2005	12	Sentences	Passive	Sentences > word lists (degr	54	-23	-7
						-59	-18	-1
	Humphries et al., 2006	21	Sentences,	Meaningfulness judgement	Interaction ((congruent sent	-66	-24	8
						-54	10	-8
						54	-9	-28
						66	-31	-8
						-54	-57	24
	Kristensen et al., 2013	24	Sentences	Sensicality judgement	Prosody x congruency intera	24	-58	60
						42	-48	48
						40	-38	44
						-30	-54	58
						-40	-46	56
	Longe et al., 2007	12	Word-sten	Pleasant/unpleasant judgment	Inflected nouns > noun sten	-16	-70	36
						-14	-69	24
						-26	-61	34
						-50	-62	2

	Mashal et al., 2009	15	written ser	Is sentence positive or negative	Novel metaphoric > Nonsen	-49	-38	28
						-49	-6	-14
						-57	-42	-1
						-9	-49	33
						-42	19	25
						-57	-43	25
	Mason and Just 2007	12	written ser	yes no judgement on a sentence	Semantic ambiguity > nonar	-28	12	16
						24	16	12
						26	46	16
						-8	18	6
						12	18	8
						-20	54	18
						34	30	6
						24	46	16
						-16	46	14
						-52	26	12
						24	12	10
						-12	20	12
						-24	22	2
						-22	-28	0
						46	18	10
						-12	-12	10
						42	12	-8
						-40	14	-6
						-42	26	-2
	Moss et al., 2005	15	written ser	Naming from definition (prime) / pic	Competition > Repetition	-40	24	-15
						-40	33	13
						-49	40	15
	Newman et al., 2010	14	Sentences	Target detection	Word order > inflectional m	58	24	12
						-34	18	38
						46	16	42
						-38	-64	40
						-58	-36	-10
	Nieuwland et al., 2007	22	Sentences	Passive	Referential ambiguity > coh	-6	66	-2
						2	64	20
						28	28	44
						-6	-62	30
						-10	-64	44

						-2	-64	48
						-50	-68	30
						50	-62	30
	Obleser and Kotz., 2010	16	Sentences	Passive	Positive correlation with int	62	-6	-4
						-60	-8	-6
						-46	-64	38
	Obleser et al., 2007	16	Sentences	Passive	Intelligibility effect but only	-50	-60	34
						-20	39	44
						4	56	27
						-40	24	-18
						-4	-56	12
	Opitz et al., 2007	24	Sentences	Target detection	Local violation > correct sen	-27	-23	-19
						26	-11	-21
						-59	-19	37
						-56	-36	27
						-66	-34	13
						65	-27	14
						37	8	-26
						-37	-10	-10
						42	-6	-11
						29	10	-9
	Peelle, Troiani and Grossman 2009	25	written sen	Sequential feature verification	Complex nouns: Inconsisten	-44	-66	30
						-50	-18	-14
						0	-40	40
						44	-68	34
						60	2	18
						-32	58	6
						0	48	-6
						-48	30	18
						-48	30	18
						-48	38	-14
						30	58	2
						2	-30	40
						-4	-66	30
						-52	-16	-20
						-42	-68	30
						0	-26	30
						40	-62	48

						10	-50	10
						-48	-38	-8
	Rapp et al., 2004	15	written ser	positive or negative connotation o s	Metaphor > literal meaning	-41	39	-7
						-48	-9	-36
						-56	-61	0
	Rodd et al., 2005	15	auditory se	Ambiguity resolution /semantic relat	Ambiguous > unambiguous	-50	-48	-10
						-52	28	20
						52	34	14
						52	22	12
						-56	-56	0
						-64	-48	-2
						-38	-40	-15
						-40	12	32
						32	26	2
	Shibata et al., 2007	13	written ser	understand the sentence? Yes or no	Metaphor > literal meaning	-48	-48	45
						-3	58	22
						1	59	13
						1	68	4
						-44	28	9
	Skosnik et al., 2002	23	Letter strir	Artificial grammar learning	AGL correct > incorrect	49	-57	42
						-24	30	52
						-52	-57	41
						-44	-76	45
						2	-62	46
	Snijders et al., 2009	28	Words/Ser	Visual catch trials	Sentence > words	-54	18	-30
						-56	-6	-16
						-62	-44	-2
						-52	34	-8
						-58	-56	12
						-58	22	12
						-44	-58	18
						-26	-6	-20
						-44	-16	-30
						-26	-36	-26
						-18	2	4
						54	20	-32
						56	8	-26
						52	-14	-16

						62	-42	0
						56	36	-10
						60	34	4
						62	28	10
						-4	54	-20
						-8	60	28
						-38	-2	-50
						-6	-62	2
						-12	-46	34
						30	-32	-32
	Stringgaris et al., 2007	11	written ser	Sentence plausible or not	Metaphor > literal meaning	-30	-64	-30
						16	-86	-11
						-6	-14	1
						-46	32	-9
						36	-68	18
						-21	-83	26
						29	-66	29
						-54	-7	37
						-29	-57	49
	Tyler et al., 2008	15	Word-sten	Pleasant/unpleasant judgment	Verb phrases > (noun and ve	30	-88	6
						-46	-36	-6
						-52	18	28
						-12	-48	40
						-2	-76	22
	Yokoyama et al., 2006	36	Sentences	Semantic plausibility judgment	Passive > active	-28	-74	46
						-52	34	14
						-52	20	30
						-32	18	-4
	Yokoyama et al., 2007	20	Sentences	Probe questions	Passive > active	-62	0	3
						-50	-62	54
	Zemplini et al., 2007a	15	written ser	Ambiguity resolution /semantic relat	Ambiguous > unambiguous	-48	26	20
						-52	16	26
						34	20	-10
						-50	-48	-12
						56	-34	-16
	Zemplini et al., 2007b	15	written ser	semantic relatedness	Figurative > Literal	-54	32	-12
						-56	28	-4
						-53	22	15

						58	23	-15
						-60	-43	-6
						-61	-35	-9
						58	-27	-16
						65	-27	-11
**Top-down attention**	Arrington et al. 2000	12	Object	Object-based cuing		-31	-32	45
						-41	-22	57
						-66	-34	6
						-47	-27	-25
						-16	-34	-5
						-16	43	32
						2	45	-18
						-33	41	-7
	Corbetta et al. 1998	6	Spatial	Peripheral attention shifting		42	-41	55
						18	-64	62
						33	-75	34
						40	1	46
						50	0	37
						33	2	55
						1	10	53
						5	-4	65
						37	50	17
						52	9	16
						39	12	7
						-32	23	5
						65	-50	6
						48	-69	8
						18	-99	14
						-23	-98	23
	Corbetta et al. 2000	13	Spatial	Posner cueing	Cue	-25	-65	56
						-25	-54	53
						-23	-67	38
						-27	-76	33
						31	-56	59
						25	-62	59
						33	-72	27
						56	-56	5
	Corbetta et al. 2002	13	Spatial	Posner cueing	Delay	-26	-57	-19

						30	-54	-15
						35	-69	-16
						-49	-72	8
						50	-66	15
						-36	-89	5
						37	-91	8
						-2	-72	21
						5	-84	13
						22	-84	30
						-6	-70	0
						11	-75	-6
						-28	-79	24
						35	-76	23
						-25	-53	48
						31	-47	42
						38	-59	52
						63	-39	32
						12	-68	45
						-2	-80	32
						57	-19	41
						48	-32	61
						61	-41	12
						52	-39	7
						-19	-60	-7
						-67	-9	13
						46	4	30
						-43	-12	31
						32	18	2
						-32	18	3
						37	29	26
						-39	31	-16
						27	11	57
						61	-9	29
						38	-25	65
	Hahn et al., 2006	23	Spatial	Posner cueing	Cue predictability increase	-31	-52	47
						-35	-48	58
						-45	-32	44
						3	-60	53

						-24	5	53
						26	2	50
						-40	31	33
						-32	-91	9
						30	98	-19
	Hill et al., 2010	16	Spatial	Cue to attend to pitch or location	cue (location) > rest	-18	-74	54
						-34	-50	48
						-30	-6	54
						24	-8	54
						-40	2	34
						-58	-58	-12
						-4	8	64
						-38	-52	48
						-12	-72	56
						-46	16	28
						-32	-2	62
	Hopfinger et al. 2000	6	Spatial	Posner cueing	Cue > target	-16	48	36
						-52	32	16
						-32	16	48
						20	8	48
						-12	-56	56
						8	-40	56
						-44	-64	32
						-44	-48	32
						44	-60	36
						36	-44	32
						0	-40	44
						4	-44	32
						-56	-24	8
						48	-12	8
						-36	-76	4
						28	-80	0
						-28	-8	20
						24	-8	12
	Huettel et al. 2001	10	Object	Visual search		28	-66	-16
						22	-72	34
						-22	-34	-8
						32	32	-4

						-28	-8	54
						8	6	54
						16	66	-10
						48	2	30
						-42	16	26
	Jack et al. 2007	4	Spatial	Delayed saccade task		-30	-55	54
	Kastner et al. 1999	5	Spatial	Posner cueing	Expectation > control	-27	-78	46
						-17	-60	62
						19	-57	65
						-38	-27	65
						43	-29	61
						-43	7	50
						40	-2	57
						1	7	63
						-31	40	26
						36	40	35
	Kincade et al. 2005	20	Spatial	Posner cueing	Cue	-39	-47	57
						-23	-54	62
						-12	-56	58
						-27	-58	40
						-39	-47	52
						-19	-57	60
	Liu et al., 2003	14	Object and	Attention shifting		26	-7	49
						-24	-10	49
						31	-50	52
						-28	-48	47
						54	-65	0
						-47	-68	5
						52	-51	7
						-44	-53	9
	Pessoa et al. 2002	9	Spatial	Working memory	Delay	3	14	48
						-25	-3	50
						29	-1	49
						-39	34	26
						-22	-59	50
						21	-65	52
						-39	-40	44
						43	-34	38

						-33	-82	14
	Pessoa et al., 2009	20	Colour	Attention switching	Switch > non-switch	-38	-82	-6
						36	-83	0
						-30	-59	-9
						-62	-46	-7
						-26	-66	39
						34	-57	44
						-36	15	29
						-21	7	59
	Rowe et al. 2000	6	Spatial	Working memory	Delay	28	11	54
						-22	16	61
						30	-56	72
						49	-30	45
						-22	-58	69
	Sapir et al. 2005	4	Spatial	Visual orienting	Cue	-42	-53	56
	Schluppeck et al. 2005	4	Spatial	Delayed saccade task		-21	-75	50
						-17	-69	61
	Shomstein and Yantis, 2004	11	Letters and	Shift>hold		4	-58	51
						-45	-44	54
						22	3	57
	Shulman et al. 1999	7	Spatial	Posner cueing	Cue	-25	-74	28
						33	-75	34
						35	-70	26
						-29	-53	64
						-32	-50	55
						-30	-47	45
						27	-60	37
						29	-54	54
						33	-46	46
						-12	-64	65
						-23	-69	54
						-12	-76	55
						20	-64	62
						25	-63	53
						29	-70	47
						-27	-3	54
						-43	-3	50
						-49	5	43

						42	-3	53
						3	4	55
						5	14	46
	Shulman et al. 2002	19	Spatial	Posner cueing	Cue	-31	-62	60
						-43	14	24
						-49	17	37
						50	-11	56
	Shulman et al. 2003	22	Spatial	Visual search		50	-67	3
						-19	-61	51
						-27	-52	55
						31	-50	56
						-25	-74	32
						-27	-9	53
						29	-7	54
						-43	-3	48
						-47	-1	30
						46	4	30
						-6	4	31
						9	6	55
						9	11	41
						11	23	33
						-30	20	-2
						35	20	2
	Silver et al. 2005	4	Spatial	Attention mapping		-23	-75	47
						-19	-73	57
						27	-75	46
						23	-73	56
	Swisher et al. 2007	20	Spatial	Attention mapping		-23	-72	48
						-21	-66	61
						-25	-58	63
						-26	-54	62
	Vossel et al., 2012	26	Spatial	Posner cueing	Valid > baseline	-29	-49	45
						29	-53	50
						-29	-6	51
						35	-2	51
**Executive semantic decisi**	Allen et al., 2008	15	written ser	Sentence completion	Inappropriate > appropriate	-44	30	30
						-42	38	32
						8	-70	50

						-4	-70	54
						-24	32	-18
	Amunts et al., 2004	11	spoken wo	Category Fluency	Category > Rote Fluency (da	-4	12	40
						4	20	24
						-42	26	22
						30	40	30
						-24	-2	54
	Assaf et al., 2006	18	written wo	object recall from feature pairs (hon	Recall trials > No recall trials	-44	14	41
						-45	35	-15
						40	25	-18
						-56	-46	38
						-66	-52	-4
						-5	-5	-1
						17	12	3
						-12	46	-2
						-8	-36	38
						-40	-47	50
						-66	-55	-4
	Badre et al., 2005	22	written wo	Semantic relatedness	Weak > Strong association	-48	-48	3
						-51	27	-3
						-48	30	-12
						-48	-48	3
						-51	15	33
	Bedny et al., 2008	20	written wo	Semantic relatedness	Inconsistent + Consistent > (	-54	9	39
						-3	33	42
						36	-69	-39
						-24	57	24
						-12	-6	18
						-45	-57	54
						-57	-42	-3
						0	27	42
						39	-66	-39
						-48	15	9
						-51	6	45
	Bunge et al., 2005	20	written wo	Semantic related Yes/No?	Low > High probe target ass	-57	24	6
	Chan et al., 2004	8	written wo	Silent generation of associate	Semantic ambiguity > non-a	-37	59	26
						-10	68	18
						-47	48	21

						-39	-17	67
						24	2	64
						49	34	15
						12	67	19
						6	39	-22
						37	-17	46
						49	-59	45
						-14	-92	18
						-52	-74	-5
						14	-93	45
						10	-75	14
						7	-82	-6
						7	22	-17
						6	-30	26
	de Zubicaray et al., 2000	8	written wo	Hayling variant (generate either an a	Inappropriate > appropriate	1	51	-17
						2	36	39
						4	47	-24
						-12	43	22
						-17	-56	48
						17	-74	44
						-2	-69	12
						-2	-71	-12
						36	-74	-18
	de Zubicaray et al., 2001	8	pictures &	Picture naming	Semantically related > unrel	-60	-21	-17
						68	4	-24
						-48	-46	19
						12	68	-9
						-4	46	-14
						-32	65	-10
						21	37	56
						-57	-31	44
						10	-81	-12
	de Zubicaray et al., 2006	13	pictures &	competitor priming paradigm	Related > unrelated blocks	-60	-45	6
						-3	33	24
						3	36	27
						-45	33	-15
						45	33	-12
						-42	18	5

						-54	-15	-18
						-48	27	30
						-21	57	18
						-6	24	48
	Dobbins et al., 2004	16	pictures &	Comparison is items smaller/larger t	Shift cue > consistent cue di	-45	6	27
						-24	-57	-10
	Gennari et al., 2007	17	written wo	General knowledge question about t	Ambiguous > unambiguous	-52	-61	5
						-44	21	-4
						-35	-43	48
						-50	4	13
	Gurd et al., 2002	11	spoken wo	Category Fluency	Category > Rote Fluency (da	-3	25	38
						10	26	31
						-33	45	23
						38	53	19
						-38	24	-17
						42	28	-19
						-37	8	27
						9	-88	-43
						-15	-65	69
						32	-60	66
	Hirshorn and Thompson Schill, 2006	10	written wo	Category Fluency	Switching > Free generation	-42	3	66
						-18	48	-6
						-3	-57	63
						-57	-57	54
						-3	-66	60
						-3	-72	0
						48	24	54
						48	24	42
						48	42	30
						3	51	-15
						39	57	3
						36	42	-9
						12	-27	-27
						42	-51	45
						-12	21	63
						-30	54	18
						-51	45	9
						-54	21	39

						-33	18	51
						-39	-3	51
						15	54	-3
						-33	27	6
						-39	-24	33
						-33	-63	21
						-18	-21	15
						-39	-39	36
						-39	-66	39
						-12	-66	51
						39	45	39
						36	51	15
						15	33	48
						66	-33	21
						51	-30	-18
						36	-54	42
	Hocking et al., 2009	18	pictures	Picture naming	Semantically related block >	-66	-30	12
						-66	-12	3
						-54	-36	15
						-30	-3	-30
						33	-6	-33
	Ketteler et al., 2008	12	written wo	Semantic relatedness	Ambiguous > unambiguous	-12	48	38
						21	45	16
						-48	39	-27
						-51	18	6
						43	39	-28
						50	-51	43
						-52	-37	58
						-31	-16	-16
						-21	41	49
						-2	-15	10
						-47	36	22
						-66	-43	-6
						50	33	18
						-2	-39	2
						41	-60	44
						-2	-29	6
						-58	26	4

						-54	20	32
						-58	20	0
						-52	18	28
	Lee and Dapretto 2006	12	spoken wo	Semantic relatedness	Metaphor > literal meaning	55	26	23
						-48	-58	56
						66	-7	-12
	Nagel et al., 2008	14	written wo	Verb generation	High > low selection	-51	26	-4
						-46	-39	46
	Nelson et al., 2009	17	written wo	Verb generation	Many > Few associates	-7	18	40
						0	11	50
						-45	0	50
						-52	22	20
						-56	11	20
						44	40	32
						44	14	8
						-32	62	-8
	Noppeney et al., 2002	12	spoken wo	Semantic categorisation	Semantic decisions conjunct	-43	24	-33
						-25	27	-34
						4	29	40
						16	-93	-31
						35	-83	-33
						-32	21	-46
						-39	5	-54
	Noppeney et al., 2004	15	written wo	Associative judgements	Different > Same triads	-44	32	10
						-44	-64	-18
						-57	-63	-1
				Synonym judgement	Difficult > Easy judgements	-32	20	-13
						-5	32	46
						-9	-5	-1
						-44	14	25
						1	-64	-49
	Ochsner et al., 2009	16	written wo	Eriksen flanker : decide is stimulus is	Incongruent > Congruent (i.	-36	22	28
						32	-6	44
						10	-10	58
						14	40	30
						-56	20	24
						36	22	16
						-38	-6	42

						34	2	28
						58	-10	44
						-16	12	50
						-16	14	34
						-8	22	46
						-6	-6	40
						-40	-66	50
						-38	-26	-16
						-32	-66	36
	Persson et al., 2004	22	written wo	Verb generation	High > low selection	-49	26	15
						41	15	5
						-52	-52	-5
						-4	8	60
	Race et al., 2009	26	written wo	Attribute judgement (small / organi	Different attribute > Same a	-51	36	12
						-48	33	0
						-45	15	24
						-51	12	15
	Roskies et al., 2001	20	written wo	Semantic relatedness (yes no decisio	Hard > Easy category verific	-54	23	-8
						-43	46	1
						17	-92	-25
	Snyder et al., 2007	14	written wo	Comparison (global vs. feature)	Semantic specific > Semanti	-24	-102	-12
						-51	-57	-18
						-57	-48	-3
						-27	-69	48
						-39	-45	51
						33	-48	51
						-54	27	24
						-36	42	0
						-57	15	6
						54	27	24
						-60	9	39
						42	18	48
						0	45	57
	Spalek et al., 2008	21	pictures &	Picture word interference	semantically related distrac	-35	-47	-16
						-51	41	2
	Thompson schill et al., 1997	6	written wo	Verb generation	High > low selection	-50	13	29
						-3	18	44
						-40	20	28

						-40	13	33
						46	21	31
						-3	19	53
						-52	-55	2
						-40	-64	-14
						-31	-71	41
						-47	9	29
						42	20	18
						-43	34	2
						-3	45	29
						-3	19	53
						-52	-59	-7
						-35	-66	53
	Thompson schill et al., 1999	8	written wo	Attribute generation: same (colour/	Different attribute > Same a	-46	20	19
	Tremblay et al., 2006	12	written wo	Category Fluency	Unconstrained > Constraine	-48	32	10
	Wagner et al., 2001	14	written wo	Semantic relatedness	Weak > Strong association	-45	27	-12
						6	18	39
						45	21	6
						54	24	27
						-57	-51	0
						-42	-51	-15
						-36	21	27
						3	30	36
						-51	21	-12
						-63	-51	6
	Whitney et al., 2009	18	written wo	Associative judgements	Ambiguous > unambiguous	-44	20	28
						-48	24	32
						-48	28	16
						-44	12	24
						-52	32	-4
						-52	20	24
						-48	8	40
	Wig et al., 2009	27	pictures &	Comparison / categorisation	Reversed or different decisi	-44	11	23
						55	9	24
	Zhang et al., 2004	14	written wo	Associative judgements on reversibl	High conflict > Low conflict	-25	23	-21
						39	20	-22
						6	37	17
						-41	23	-14

						-45	15	18
						1	25	33
						39	20	-18
**Tool-praxis decisions**	Chao et al., 1999	6	greyscale p	viewing	tools > animals	29	-50	-10
						-27	-49	-5
						44	-55	0
						-48	-57	5
				covert naming	tools > animals	29	-50	-18
						-28	-53	-16
						55	-55	7
						-47	-59	10
	Chao and Martin, 2000	5	greyscale p	viewing	tools > animals	-31	20	4
						-53	7	24
				covert naming	tools > animals	-44	10	21
						-33	-44	47
						-31	-35	52
	Chao et al., 2002	7	greyscale p	covert naming	tool > finger tap	-26	-60	-6
						25	-56	-5
						-46	-57	4
						-32	-44	50
	Creem-Regehr and Lee, 2005	12	3D images	imagining grasping tools	tools > faces	-46	-52	-13
						-35	-47	-18
						57	-62	2
						48	-67	1
						42	-44	-19
						53	-59	22
						-43	-19	57
						32	-37	63
						-56	5	23
						-7	29	41
	Downing et al., 2006	12	colour pho	viewing	action > action-irrelevant se	8	-27	8
						-18	-44	17
						-32	-40	46
						-41	-30	45
	Gerlach et al., 2000	15	line drawir	categorization	tools > faces	-47	40	-30
						-32	55	-30
						-34	46	-31
						-57	41	10

						-62	29	-6
						-68	10	18
						-11	22	36
	Grafton et al., 1997	8	visual imag	viewing	tools > objects	-40	0	53
						-33	50	10
	Grezes and Decety, 2002	10	colour pho	orientation judgment	tools > objects	14	27	19
						47	51	16
						51	-67	19
						64	27	-13
						53	-29	-9
						31	-1	-14
						55	-5	-27
	Handy et al., 2003	14	line drawir	viewing	tool > rest	-12	27	31
						56	25	26
						-46	15	46
						-3	25	33
						59	-49	35
						-56	-49	37
	James et al. 2002	6	3D images	viewing	tools > non-meaningful mov	38	-46	-22
						-36	-50	-24
						29	-74	41
						-12	-71	45
	Moscovitch et al., 1995	13	line drawir	1-back task	tool > sham arm movement	30	-71	24
						28	-59	37
						30	-54	45
						44	-55	-18
						46	-57	-13
						44	-63	-4
						44	30	19
						8	-42	-14
						-3	-42	-5
						42	0	-19
						-53	-48	19
						1	52	-19
						3	61	-6
						8	51	17
	Price et al., 1996	6	coloured d	viewing	tools > scenes	-51	-25	-33
	Robert et al. 2010	14	visual imag	categorization	tools > objects	-52	-70	-1

						53	-67	-7
						42	-90	-1
						60	-40	9
						-47	13	7
						10	-2	-12
	Spiridon et al. 2006	14	greyscale p	1-back task	tools > faces	32	-51	-13
	Vingerhoets, 2008	15	colour pho	orientation judgment	tools > objects	-54	-57	37
						-5	-48	25
						-7	-69	41
						-57	-38	1
	Wadsworth and Kana, 2011	32	colour pho	imagining tool use	tools > rest	21	-95	6
						-50	12	30
						27	-27	-3
						-20	-30	-3
						61	42	11
						70	20	6
						-20	6	1
						48	12	22
						-16	-5	-4
						-35	17	-8
	Kassuba et al., 2011	19	greyscale p	1-back task	tools > scrambled	-50	-27	16
						-44	-8	0
						-40	-27	19
						-34	29	-5
						-63	4	-7
						65	-15	12
						62	-27	14
						56	7	-9
						46	-5	-7
						43	-3	8
						-44	-49	-5
						-41	-47	-14
						-47	-71	-2
						-28	-48	-17
						-34	-30	-22
						-25	-53	-13
						-44	39	-3
						59	38	-14

						46	39	-11
						65	21	-3
	Wiggett et al., 2009	16	greyscale p	categorization	tools > animate objects	34	-46	-6
						-28	-45	-9
	Anzellotti et al., 2011	12	greyscale i	categorization	tools > animals	-27	-41	-21
						27	-49	-15
						-46	-63	-10
						-55	-14	-32
						-37	-11	-39
						-47	3	-41
						-25	-78	38
						-38	-40	38
						-46	-31	37
						54	-29	38
						-31	-50	50
						-46	12	31
	Damasio et al., 1996	9	photograp	overt naming	tools > fixation	-55	-53	-10
						-48	26	4
						-4	42	25
	Grabowski et al., 1998	9	photograp	overt naming	tools > objects	-46	27	3
						-29	59	8
	Grafton et al., 1997	8	visual imag	covert naming	tools > scrambled	-1	11	65
						-40	0	53
						-33	50	10
						-37	49	3
						-40	21	13
						20	32	22
	Grezes and Decety, 2002	10	colour pho	covert naming	tools > rest	-59	-1	46
						-33	45	30
						-51	19	6
						-59	-30	2
						-38	-32	-19
	Kiyosawa et al., 1996	4	visual imag	overt naming	tools > rest	36	4	-37
						-35	-1	-35
						34	38	-36
						-37	42	-35
						-41	50	-22
						6	74	-21

						-20	73	-11
						43	-27	-3
						-56	-36	4
						-24	-7	6
						-41	37	19
						33	6	30
						-51	1	32
						-12	-19	42
	Liljestrom et al., 2008	15	line drawir	covert naming	tools > non-meaningful obje	-50	-73	6
						-33	-53	-19
						-35	-43	-24
						-30	-56	52
						-50	-30	44
						-44	15	6
						-36	34	-13
						42	-50	-17
						44	-84	12
						23	-64	52
						32	-51	52
						38	-87	16
	Martin et al., 1996	16	line drawir	covert naming	tool > objects	-29	-53	-12
						38	-53	-13
						-38	-50	-3
						-25	6	4
						-12	-17	6
						-31	11	4
						-38	3	9
						31	32	-4
						10	-61	-30
	Okada et al., 2000	12	greyscale i	covert naming	tool > objects	-48	30	16
						-48	-36	56
						-40	-34	44
						-50	-52	-8
						-46	-68	-4
	Tranel et al., 2005	10	colour pho	covert naming	tools > animals	-27	-31	-18
						-53	-49	-15
	Valyear et al., 2007	11	greyscale p	covert naming	familiar tools > unfamiliar to	-46	-33	49
						-51	-62	7

						-49	17	10
	Wierenga et al., 2009	20	greyscale p	covert naming	tools > animate objects	-54	-57	0
						48	-73	4
						-60	-30	39
						37	-36	44
						-27	-72	37
						-50	-69	28
	Johnson-Frey et al., 2005	24	names (a	planning tool-use gestures	tools > scrambled	-43	-48	57
						-33	-66	63
						-44	39	-7
						-41	36	-20
						-32	37	-21
						-53	11	22
						-9	52	33
						-44	40	-7
						-54	22	-2
						-52	-56	-5
						-63	-37	1
						-33	-29	0
						-42	-2	-4
						71	-25	-2
						30	5	6
	Creem-Regehr et al., 2005	12	3D images	viewing	tools > shapes	-44	-75	2
						-42	-36	-15
						48	-62	6
						48	-40	-16
						50	-67	-1
						44	-48	14
						-41	-44	63
						-19	-53	68
						-26	0	58
						28	2	57
						-9	13	60
						47	-30	66
						-55	7	4
						15	16	51
						-16	12	-2
	Kellenbach et al., 2003	9	coloured p	manipulation judgment	tools > control	-48	-64	-5

						-44	-29	-24
						-48	8	16
						-38	34	-24
						-44	40	-12
						-58	-14	23
						-39	-37	47
						-58	-41	23
						28	80	-57
	Choi et al., 2001	10	names	planning pantomimes	tools > scrambled	-37	-44	63
						-15	-5	68
						-2	12	66
						-3	-95	-22
						6	-63	-3
						1	-72	-11
						-59	-60	-16
						-16	10	-5
	Boronat et al., 2004	15	line drawir	manipulation judgment	tools > scrambled	-55	11	10
						-3	26	33
						-34	1	48
						-34	-70	48
						-55	-7	-12
						50	-83	7
						-19	-2	1
	Hermsdorfer et al., 2007	23	video clips	planning tool use	planning tool use > watchin	-47	-59	15
						59	-55	12
						-37	-32	51
						-50	-23	47
						47	-22	51
						63	-37	28
						-33	-39	63
						-20	-52	63
						-21	2	64
						31	12	68
						-31	29	-11
						-50	11	24
						-34	42	18
						-34	50	26
						-8	20	36

						-25	7	-11
						30	5	-6
	Ohgami et al., 2004	18	names	planning pantomimes	planning tool use > animal r	2	18	53
						-26	9	67
						-46	-38	60
						-30	-51	73
						-51	13	23
						-46	40	17
	Fridman et al., 2006	19	written co	plannning transitive gestures	planning tool use > pattern	-42	25	15
						-56	11	32
						-44	11	24
						10	13	45
						-3	17	34
						-5	4	41
						-65	-30	28
						-61	-26	23
						8	-32	23
						-5	-34	28
						8	-41	18
	Bohlhalter et al., 2009	15	written co	planning transitive gestures	planning tool use > rest	37	26	-11
						-40	29	-2
						6	20	38
						-38	-14	-53
						36	-73	43
						-32	-65	45
						65	-51	-7
						-59	-57	-16
						44	-56	-31
						-50	-56	-22
	Wadsworth et al., 2011	32	colour pho	viewing	tools > animals	-12	-88	-2
						-50	15	19
						-26	-53	70
						-22	-29	0
						-9	19	61
						-28	12	54
						27	-29	-1
						36	-65	42
						-18	-5	-3

	Phillips et al., 2002	26	pictures/pl	manipulation judgment	manipulation judgement > c	-51	22	12
						-47	38	-3
						-59	-65	1
						-55	-40	-11
						-45	-11	-43
						-49	-39	-25
	Canessa et al., 2008	15	colour pho	manipulation matching	manipulation judgement > s	-32	-48	59
						-52	-23	43
						-26	14	54
	Vingerhoet et al., 2009	15	3D images	imagining grasping/using tools	use tools > move tools	-25	-49	72
						-33	-39	57
						-44	-26	51
	Peran et al., 2010	12	line drawir	imagining tool use	imagine tool use > size judg	-22	8	57
	Grafton et al., 1996	7	real tools	imagining grasping tools	imagine grasping > scramble	-5	0	60
						-19	-9	62
						21	-16	57
						1	5	50
						-50	-52	43
						-25	-60	39
						-21	-75	38
						-45	4	29
						-44	31	24
						15	17	7
						-51	-11	5
						37	-63	-34
	Buxbaum et al., 2006	15	pictures	action decision	Prehensile use > scrambled	-60	-27	43
						-50	5	33
						-5	-25	-12
						31	-60	65
						41	-5	59
**Default mode network**	Buckner, 1998	26			Recognition - Fixation, Decr	7	59	3
						8	-48	31
						4	39	8
						14	52	-3
						-1	-49	52
						2	-47	38
						62	-55	20
						5	-16	48

						65	-6	15
						2	-26	49
						4	50	4
						48	-71	30
						-42	-76	24
						62	-37	43
						-6	52	-3
						-59	-26	16
						-6	52	-3
						-21	27	49
						44	-14	-1
						-56	-10	11
						-39	-14	1
						-1	-45	66
						-8	68	27
						25	-73	50
						-36	1	-7
						-2	57	25
						68	-20	1
						-17	-38	69
						-45	-22	19
						-56	0	13
						65	-11	33
	Sadato, 1998	8			Rest-Discrimination (Sighte	-18	24	56
						-12	57	-6
						-23	62	3
						34	-89	-10
						49	-73	2
						-25	-99	6
						-7	-104	-8
						21	-74	-7
						-4	-40	62
						19	-42	58
						1	18	-15
						-49	36	-3
	Rosen, 1999	13			Rest > Endogenous	6	-70	37
						11	-29	75
						-1	-91	7

						5	-88	11
						-5	-56	22
						-58	-8	8
					Rest > Exogenous	0	-74	34
						6	-73	33
						-1	-88	13
						6	-78	50
					Rest > Control	-1	-77	41
						6	-77	40
						12	-39	74
						5	-89	9
	Fox, 1996	10			Rest > Solo, Controls	-27	20	-11
						16	40	-4
						14	51	-3
						-21	53	-1
						53	48	-17
						-27	16	41
						-24	-17	-17
						-38	-3	-5
						12	29	15
						10	41	9
						-16	21	43
						31	3	-12
						40	49	-1
						53	47	-8
						-16	35	30
					Rest > Chorus, Controls	16	38	-4
						-21	55	-3
						-16	35	30
						7	28	-19
						-38	-5	-6
						-6	12	-8
						12	45	4
						8	-36	32
						16	-40	6
						-16	22	37
						-25	26	-11
	Sadato, 1996	8			Rest > Discrimination, Sight	-5	-104	-12

	Petit, 1999	10			Rest > DIS	-44	-30	12
						44	-30	12
						-52	-4	4
						50	2	4
						-44	12	-16
						42	14	-16
						-54	-62	20
						54	-64	16
						-36	10	48
						-34	20	32
						-4	46	20
						12	36	40
						-4	-4	36
						-2	-72	28
						-12	-46	8
						6	-58	16
					Rest > FIX	-40	-32	8
						44	-30	12
						-56	-4	4
						50	2	4
						-42	4	-16
						44	-2	-16
						-54	-64	20
						50	-72	20
						-54	24	20
						22	58	-4
						-10	-58	32
						2	-62	32
	Rajah, 1998	12			LV 2, Deactivations	14	-84	-28
						44	-61	-31
						59	-31	-29
						10	19	2
						44	-6	-14
						64	-55	4
						62	-32	42
						6	-25	38
						-66	-58	11
						-59	-41	27

						-61	-55	38
						-59	-58	-16
						-27	-51	-17
						-29	9	-18
						6	-88	0
	Tzourio-Mazoyer, 1998	5			Rest > Text in Right-Handers	42	46	10
						42	50	-2
						50	34	26
						16	-54	20
						36	-72	20
						30	-74	34
						54	-48	-8
						50	-38	-20
						44	-32	-20
						62	-26	28
						-8	-70	36
						8	-68	40
						14	68	-2
						-58	-22	32
					Rest > Text in Left-Handers	-58	-28	34
						-50	-44	46
						-36	-36	48
						-50	-60	-10
						-10	-58	66
						4	-36	48
						-8	-34	52
						-44	30	32
						-36	32	20
						-38	46	8
						62	-32	34
						50	-52	48
						54	-38	42
						-22	2	66
						38	28	36
						26	8	60
						56	-36	-22
						48	42	8
	Muley, 2001	10			Baseline - Activation Effects	3	35	-15

					Baseline - Activation Effects	0	-41	45
						1	-51	44
	Shen, 1999	9			Common Deactivation	-23	-54	-4
						-51	-7	0
						-9	-43	36
						1	-11	46
						-9	48	-19
						53	-7	-1
						3	-11	46
						10	48	-20
	Brannan, 2001	9			Rest - CO2 FM	-1	26	31
						2	-23	31
						2	-49	25
						33	52	-19
						-34	50	-18
						-25	55	-1
						0	10	-26
						-24	28	47
						-31	14	42
						34	27	41
						-58	-19	-33
	Fiez, 1999	11			Fixation - Word Reading	28	59	5
						15	55	-18
						28	53	1
						45	53	5
						41	33	25
						-38	26	38
						35	28	35
						-27	8	53
						31	2	53
						29	-47	35
						5	-41	37
						7	-43	60
						-1	-63	51
						-14	-70	43
						16	-58	55
						44	-45	55
						-36	-60	42

						57	-54	33
						48	-40	46
						35	-71	38
	Jernigan, 1998	8			Fixation > Word Identificati	4	-37	44
						58	-28	20
						5	39	-17
						21	26	51
						-59	-36	31
						51	-69	19
						-11	-68	61
						-19	0	-18
						22	-2	-23
						-18	15	48
						-42	-73	26
					Fixation > Word Recognition	58	-28	20
						-59	-32	31
						5	39	-17
						-46	-68	16
						49	-71	20
						-16	-2	-22
						-29	23	43
						25	-2	-23
						-35	38	19
	Rumsey, 1997	14			Fixation - Irregular Pronunci	45	-71	20
						-44	-69	21
						4	-56	41
						62	-40	30
						58	-34	29
						4	-28	47
						46	-22	-30
						31	-17	-21
						-25	-13	-21
						-49	-9	-25
						-45	-1	-26
						-21	4	-27
						-27	21	43
						25	27	42
						38	30	19

						-21	17	-19
						-23	43	-17
						1	31	-35
						8	50	8
						-10	45	-22
						-23	51	-5
						-12	54	8
						9	55	-10
						4	43	31
					Fixation - Pseudoword Pron	-42	-74	21
						45	-71	20
						13	-56	50
						58	-50	35
						43	-32	29
						53	-29	29
						-52	-44	37
						21	-30	43
						-3	-50	23
						-16	-26	48
						48	-18	-31
						25	-11	-26
						-23	-5	-26
						-45	1	-31
						-24	21	43
						27	27	37
						5	33	-35
						-25	49	0
						-14	47	-22
						8	52	13
						7	52	-19
					Fixation - Phonological	51	-69	19
						-46	-67	25
						-42	-67	25
						60	-47	26
						58	-26	24
						-5	-50	18
						-5	-31	43
						53	-21	10

						-29	-5	-26
						46	-11	-27
						46	-10	-18
						-45	0	-22
						-24	29	38
						-1	55	12
					Fixation - Orthographic	51	-69	19
						-46	-63	25
						62	-47	26
						62	-44	30
						58	-26	24
						-7	-48	23
						-5	-31	39
						6	-53	36
						-50	-56	24
						55	-19	5
						-29	-13	-21
						48	-11	-22
						47	-72	29
						-47	-5	-21
						-34	-7	-21
						-24	25	43
						1	52	-19
						-14	56	4
						3	54	8
						-3	56	8
	Li, 2003	20			Rest > Conventional Acupur	3	-82	16
						-2	-75	14
						52	-18	12
						-52	-15	12
						-50	40	16
						50	40	12
	Ingham, 2000	4			Rest - Overt Solo, Controls	-26	16	46
						0	-57	55
						-57	-28	31
						56	-25	33
						69	-34	-16
						-1	40	14

						1	41	0
						-38	-4	-10
						44	-16	-8
						-23	7	-13
						30	3	-12
					Rest - Imagine Solo, Control	0	-56	48
						51	-6	4
						-55	-15	-18
						55	-43	17
						-1	-51	32
						5	37	-16
						-42	-13	6
					Rest - Overt Chorus, Contro	-2	-10	64
						-29	15	46
						11	-39	64
						58	-26	33
						-55	-40	-11
						62	-54	22
						-10	42	-9
						5	37	10
						-42	20	-1
						44	18	-12
						27	-94	-18
						-12	-40	-7
					Rest - Imagine Chorus, Cont	-2	-21	57
						-13	-34	57
						-3	-47	25
						5	35	-15
						-21	8	-5
						-22	-34	-27
	Beauregard, 1997	10			Baseline - Random Letters	1	17	38
						67	-37	21
						5	-48	50
						1	-56	34
						4	-55	56
						56	-54	14
						-3	-56	64
						11	-49	-11

					Baseline - Concrete Words	11	-25	39
						-5	-58	58
						-41	-47	-35
						-3	-3	39
						7	-39	47
						5	-44	48
						-27	-5	72
						1	-74	31
						-38	-21	9
						-5	-68	42
						-40	-43	41
					Baseline - Abstract Words	-41	-44	-36
						56	-32	39
						13	-24	39
						-50	-39	39
						67	-40	21
						5	-41	47
						-47	-41	40
						-16	-44	-18
						-25	-8	72
						-3	-56	58
						-60	-39	34
						-60	-37	30
						-11	-67	49
					Baseline - Emotional Words	-37	-45	-35
						11	-24	39
						60	-25	17
						44	-41	38
						9	-32	45
						63	-29	38
						-36	-41	41
						-44	-11	8
						-47	-8	8
						-60	-32	27
						-1	-74	33
						21	-64	20
						-5	-73	34
						-3	-71	34

						5	-48	50
						5	-44	48
						52	-66	11
						60	-28	27
					Baseline - Random-Letters I	11	-24	41
						-43	-45	-35
					Baseline - Word Instructions	-44	-42	-35
						23	-100	-14
						55	-50	0
						43	-25	-35
						50	-34	-32
	Gemar, 1996	11			Rest - Sad Mood	44	52	7
		11			Rest - Neutral Recall	49	40	17
						-21	-91	-27
	Tatsumi, 1999	6			Rest - Verb Generation, Poo	60	-24	24
						15	-64	33
						12	64	-7
	Fiez, 1995	10			Fixation - Auditory Target D	-21	-76	32
						28	-71	33
						25	-49	50
						-42	-22	44
						56	-25	40
						-42	-66	17
						45	-54	21
						42	-74	19
	Fiez, 1996	12			Negative Changes in Short T	9	-18	51
						19	-3	-9
						-14	-47	32
						39	-21	-3
						-26	-14	-12
						48	-5	0
						26	6	8
						51	-13	57
						-47	-19	3
						43	-14	27
						29	-31	25
						-54	-86	16
						44	-53	20

						39	-84	14
						-7	-20	58
						5	-54	30
						65	-42	25
						-50	-21	61
						-34	-32	19
						39	-101	9
						-30	-95	5
						-32	-82	31
						18	-61	3
						-23	-33	48
					Negative Changes in Silent (	-17	43	-6
						-34	24	-2
	Nunneley, 2002	9			Rest - Heating	-23	-2	11
						17	-61	12
						12	-102	10
						27	-87	-12
						-35	-33	21
						-44	-23	16
						19	-71	26
						-46	-35	20
						-14	-98	-10
						-25	-12	11
						29	-10	12
						-13	-75	28
						-14	3	18
						6	-90	21
	Bodegard, 2000	6			Rest > Somatosensory Discr	-34	-82	24
						-18	-85	22
						0	-66	17
						26	-83	21
						36	-80	13
	Mirz, 1999	5			Continuous White Noise > B	1	-66	33
					Continuous Pure Tone > Bas	1	-62	30
					Tone Pulse Trains > Baseline	-1	-51	51
						60	-52	38
						44	-65	35
						-4	-29	49

					Tone Pulse Trains, Baseline	1	-69	33
						15	-35	69
						-4	-27	43
					Single Words > Baseline, De	1	-47	47
						-3	-34	46
						58	-50	34
						43	-44	-14
						-42	-71	33
						28	36	52
					Standardized Speech > Base	11	-64	53
						53	-59	-3
						-7	-56	29
					Classical Music > Baseline, D	58	-43	36
						-11	-41	48
						1	-68	36
						-7	-39	48
						49	-61	34
	Murtha, 1999	10			Abstract Patterns - Baseline	-12	-69	18
						-23	-85	33
						-20	-83	31
						39	-85	40
						47	-73	-5
						-25	-54	-8
						-20	-49	-3
						52	-62	11
						64	-59	0
						-1	-56	48
						61	-24	38
	Murtha, 1999	10			Abstract Patterns - Anticipat	-3	-56	49
						-5	-55	49
						-5	-60	48
						-5	-67	51
						-11	-56	48
						-5	-67	55
						-4	-60	57
						-9	-74	-59
						-1	-58	57
						21	-57	35

						27	-64	42
						68	-18	35
					Picture Naming - Baseline (D	-55	-61	15
						-59	-19	-27
						-65	-54	-8
						59	-57	-2
						56	-57	-3
						59	-26	18
						55	-64	15
						0	36	-24
						37	33	41
						-59	-39	42
						-64	-34	29
						1	-58	48
						61	-29	41
						43	-43	42
						0	-59	57
					Picture Naming - Anticipatic	-12	47	-10
						-7	49	-6
						36	37	38
						56	7	23
						4	43	-3
						-9	-17	47
						-4	-55	49
						-1	-56	45
						-4	-55	52
						-4	-53	54
						-3	-60	57
						-59	-37	42
						9	-32	39
						7	-38	21
						69	-26	27
						67	-25	30
						60	-27	38
						-61	-30	29
					Semantic Judgment - Baseli	51	19	-36
						54	-23	-25
						-1	37	-10

						35	33	43
						0	64	11
						4	64	14
						1	55	-25
						0	29	-22
						3	22	-18
						63	-45	32
						63	-44	35
						63	-37	38
						57	-26	23
						59	-56	24
						3	-56	47
						60	-52	9
					Semantic Judgment - Antici	-13	-56	6
						-1	59	7
						-3	52	-10
						-25	9	62
						1	18	-10
						3	27	-21
						33	31	43
						32	33	46
						30	31	50
						39	21	31
						13	61	-4
						1	-48	32
						0	-68	30
						1	-25	45
						1	-27	45
						55	-16	16
					Abstract Patterns Anticipati	-27	-62	-7
						38	17	-33
						36	10	4
						1	34	-22
					Picture Anticipation - Baseli	-33	-75	-35
						40	17	-30
						-5	58	-22
						7	41	-27
					Semantic Judgment Anticipa	-1	55	-25

						44	-74	-6
	Kumari, 2003	6			Rest > Pulse, Normals	-2	58	2
						-2	44	14
						8	-64	4
						-44	-20	56
						-46	-30	56
						-36	-38	62
						46	-22	54
						50	-14	52
						32	-24	50
						-62	-12	22
						-58	-8	10
						-60	-14	-8
						58	-12	34
						54	-4	34
						60	-10	22
						48	-54	18
						56	-62	24
						40	-56	18
						-42	-68	16
						-46	-74	-4
						-4	-58	16
						-2	-68	18
						4	-60	18
	Calhoun, 2001	10			Figure Selection > Fixation, [illegible]	-51	-62	40
						52	-46	26
	Anderson, 1994	8			Reflexive Saccades > Fixatio	-36	19	-19
						42	22	-16
						-27	-97	1
						30	-96	4
						-22	52	31
						20	58	7
						-3	48	9
					Remembered Saccades > Fi[illegible]	-36	-34	-14
						31	-24	-21
						-20	-101	1
						30	-96	4
						-22	50	31

						-4	39	-17
	Braver, 2003	13			Mixed State > Fixation, Decr	-42	33	-7
	Sweeney, 1996	11			Visually Guided Saccades - F	-27	32	24
						38	30	23
	Heim, 2002	12			BASE - NULL, Deactivations	-38	13	34
	Doricchi, 1997	10			Fixation > Fast-Regular	-16	25	-2
						19	18	16
						-18	-20	16
						9	2	-27
						-11	-32	53
						26	-32	52
						38	-86	-1
					Fixation > Antisaccades	-16	21	-6
						5	39	-17
						-40	-85	9
						42	-81	3
						-49	-22	-24
						46	-41	-24
						-64	-45	5
						62	-23	6
						12	4	-28
						-14	-25	7
	Bestmann, 2004	12			Rest - Suprathreshold rTMS	3	-31	61
						44	-24	58
						20	-84	30
						33	-84	18
					Rest - Subthreshold rTMS	2	-25	62
						3	-84	19
						40	-25	57
						17	-85	36
						34	-82	13
	Christoff, 2004	12			Rest > Arrows	40	12	-28
						-38	8	-20
						-14	-44	-16
						12	-40	-12
						24	-18	-36
						-40	50	20
						72	-24	28

						-38	-26	8
						24	-34	52
						-16	-60	12
						-4	-98	4
						-40	-88	16
						-6	-92	16
						-10	-68	32
						12	-98	8
	Ferrarelli, 2004	6			TMS, Left M1 - Rest, Decrea	12	-55	-20
	Hlushchuk, 2006	10			Tactile Stimulus, Right Hand	-9	-63	-14
						-32	-47	-26
						-22	-75	35
						41	-17	57
						21	-59	58
						28	-49	64
	Knoch, 2006	16			Baseline - 10 Hz Left Sided S	-26	-12	-32
						14	6	54
					Baseline - 1 Hz Left Sided St	-38	-22	-20
						22	-54	-12
						-54	38	0
					Baseline - 10 Hz Right Sided	30	68	-6
						32	16	48
						-8	-14	52
					Baseline - 1 Hz Right Sided S	-16	-32	-16
						4	-60	8
	Wicker, 2003	7			Rest > Tasks	4	44	-2
						2	32	10
						-24	46	14
						32	50	4
						22	36	34
						-28	24	42
	Meltzer, 2005	4			Encoding > Fixation, Deactiv	1	34	4
						-46	-13	5
						43	-12	5
						23	-48	8
						-24	-52	-11
	Rowan, 2004	10			Verb Generation, Deactivati	48	-72	39
						0	-51	24

						-51	-69	30
						0	30	-12
	Napadow, 2005	13			Manual, Decreases	-18	-9	-25
						19	-15	-20
						-18	-13	-24
						-5	30	-13
						-6	70	1
						3	51	-16
						-2	51	-17
						7	55	29
						-4	53	9
						33	20	-31
						-53	14	-19
					2-Hz Electroacupuncture, D	-16	-9	-25
						29	-18	-16
						4	23	-8
						6	12	-10
						5	64	-21
						-1	67	0
						3	35	-18
						-1	35	-18
						-26	16	-36
					100-Hz Electroacupuncture,	-21	-3	-27
						38	50	-15
						-35	38	-18
						2	31	-12
						-3	-50	24
						5	-11	-7
						-1	65	-18
						6	66	1
						-1	66	-2
						3	42	-21
						-1	42	-21
						44	12	-44
						-44	11	-35
	Hui, 2005	11			Deqi Only, Deactivations	28	-8	-24
						-25	-3	-25
						38	-23	-13

						-17	-17	-21
						19	-4	-36
						-27	-9	-36
						3	-1	7
						-1	-2	9
						3	4	6
						-1	4	6
						10	8	-12
						-4	11	-7
						5	34	-15
						0	32	-10
						2	37	-6
						-5	39	2
						3	47	14
						4	-12	38
						-1	-12	38
						7	-38	37
						-1	-34	36
						5	-39	41
						9	-51	16
						-4	-57	22
						-4	-52	23
						-41	9	-12
						28	14	-34
						-28	10	-38
						3	70	-23
						-22	58	-2
						5	33	-24
						-3	42	-20
						4	18	-21
						-1	6	-13
						16	16	3
						-6	16	3
						18	-29	1
						-10	-31	4
						62	-32	37
						-38	-37	52
						6	-8	57

						-2	-12	67
						13	-20	-14
						-3	-21	-19
						5	-34	-32
						-5	-32	-36
						6	-20	-34
						0	-25	-34
						1	-40	-15
						-1	-58	-1
						3	-76	-13
						-1	-76	-14
						5	-78	-36
						3	-68	-36
						10	-69	-48
						2	-59	-40
						-2	-52	-33
						1	-48	-33
						29	-35	-27
						-8	-40	-12
						17	-53	-14
						-16	-53	-13
						17	-89	-24
						-15	-92	-18
						20	-84	-34
						-12	-77	-31
						15	-85	-45
						-10	-74	-31
						12	-65	-39
						-15	-57	-59
						7	-72	-42
						-6	-60	-56
						-23	-43	-43
						41	-42	-31
						-29	-35	-28
						39	-42	-32
						-22	-47	-32
						41	-48	-34
						-38	-43	-38

						-33	-73	-45
						22	-70	-53
						-38	-50	-47
						31	-40	-43
						-37	-53	-47
						3	-54	-26
						12	-61	-30
						-20	-51	-29
					Deqi + Pain, Deactivations	37	11	-40
						-37	0	-44
						10	68	-3
						-4	65	-2
						3	15	-24
						59	-55	39
						42	-49	-45
					Sensory, Deactivations	4	33	-15
						-10	66	-11
						6	18	-21
						4	13	-18
						3	-83	-38
	Paulesu, 1997	6			Rest > Verbal Fluency	56	-69	24
						64	-47	26
						-52	-64	30
						-63	-39	27
						-61	-24	21
						-3	-56	28
						10	-66	29
						-7	-75	30
	Eyler, 2004	9			Fixation > Vigilance, Normal	30	-36	53
						-3	-27	69
						33	-5	-45
						-47	45	3
						-31	-20	48
	Warburton, 1996	9			Rest > Verb-Noun Comparis	-48	-34	5
						-51	-44	-9
						-51	-2	2
						-44	7	-7
						-40	23	-4

						62	-32	0
						57	-5	-5
						38	7	8
					Rest > Verb Generation, Gro	-40	-78	-1
						34	-61	-13
						47	-65	19
						45	-16	32
						14	46	31
						-1	-67	24
						15	-39	44
						-9	-30	48
						7	46	-9
	Smeets, 2006	12			Satiation with Chocolate in	-54	-34	54
						-42	-46	62
						0	26	46
					Satiation with Chocolate in	2	-4	-4
						-18	-6	-22
	Ghatan, 1995	8			Perceptual Maze > Rest, De	23	59	31
						8	62	9
						6	50	-21
						33	22	-21
						6	49	-16
						7	-31	30
						5	-36	30
						61	-8	9
						61	-7	12
						56	16	-24
						53	-33	22
						63	-49	27
						63	20	20
						-7	63	23
						-4	62	10
						-5	48	-20
						-58	29	7
						-53	27	-15
						-5	49	-16
						-11	-25	30
						-2	-38	31

						-58	-8	11
						-65	-22	13
						-58	-40	17
						-58	-50	26
						-60	-55	30
						-62	-12	26
					Motor Control > Rest, Decre	4	47	-9
						4	41	-9
						41	-8	9
						7	-48	41
						-7	49	-9
						-2	41	-9
						-60	-28	27
						-4	-53	42
	Pfefferbaum, 2001	10			Rest > 2-Back, Controls	-7	69	1
						-1	58	14
						8	67	-12
						9	69	2
						17	71	-2
						-8	-47	8
						-4	-61	15
						-4	-53	22
						-12	-57	25
						18	-56	31
						12	-63	31
						6	-30	59
						2	-39	69
						-6	-26	58
					Rest > Match-to-Center, Co	5	-68	38
						12	-62	33
						11	-61	9
						-12	-65	17
						-7	-69	0
						-27	-58	39
	Egan, 2003	10			Infusion > Rest, Deactivatio	6	-54	-28
						22	-38	12
						4	-8	-18
						12	2	18

						-1	-52	-36
						-2	8	6
						-20	-18	22
						-8	10	0
						-44	-44	-10
						1	-42	28
						-6	18	44
						4	32	38
						32	2	32
						12	10	-16
						21	34	6
						18	30	-6
	Denton, 1999	10			Maximum Thirst, Deactivati	18	-6	-18
						7	-14	15
						-1	-10	12
	Georgiou-Karistianis, 2007	17			Simon Task Deactivations, N	-6	38	14
						2	40	10
						-4	-36	40
						4	-42	28
	Hamdy, 1999	8			Decreased rCBF During Volit	8	52	22
						-38	6	40
						50	-14	-10
						-60	-22	2
						14	-46	72
						-16	-44	72
						62	-50	6
	Harris, 2005	8			Deactivations During Volitio	36	4	28
						2	12	-22
						60	34	0
						-20	40	-8
						-60	22	12
						46	-46	-18
						54	6	-18
						-68	-30	-10
						-36	-32	18
						-2	-44	-16
	Carlsson, 2000	7			Rest > Tickle	24	20	32
						-32	60	4

						-36	44	-8
						-48	20	36
						-28	16	48
						-40	20	24
						36	-20	40
						-40	20	44
						0	-24	48
						-8	-68	28
						40	-64	40
						-36	-52	20
						-36	-56	44
						-4	-68	48
					Rest > Anticipation	24	20	36
						-28	24	44
						28	-28	64
						-24	-24	56
						0	-16	48
						-4	-56	32
	Ganguli, 1997	8			Auditory Memory Related D	-4	51	-23
						-38	-69	3
						51	-62	1
						-3	-68	11
						21	-70	15
						-5	-46	18
						8	-65	15
						-31	-31	39
	Brown, 2006	10			Fixation > Prosaccade Respo	-3	52	27
						0	30	9
						49	-60	31
						-46	-62	33
						9	-30	71
						-3	-42	31
					Fixation > Antisaccade Resp	-2	52	32
						46	-61	32
						-50	-67	35
						-2	-48	29
					Fixation > Nogo Response	2	54	20
					Fixation > Nogo Instruction	34	-2	58

						-30	-2	64
						-53	-25	17
	Andreasen, 1995	13			Rest > Episodic Memory	20	56	-32
						42	45	-15
						29	10	52
						-52	-43	-21
						54	-45	-25
						50	-63	18
						9	-55	53
						-58	-38	36
						57	-35	39
						42	-11	-11
					Rest > Word Generation	3	48	-31
						12	48	54
						49	-7	6
						55	-42	-28
						-46	-61	22
						52	-61	18
						5	-52	-21
	Dohnel, 2008	16			All 2-back > Fixation, Norma	9	38	31
						44	-64	42
	Redcay, 2008	10			Rest > Forward Speech, Tod	-18	-7	3
						-17	-38	61
						-36	-28	56
		10			Rest > Forward Speech, 3 Ye	50	-6	25
						28	62	0
						-10	-17	2
						16	-20	6
		10			Rest > Backward Speeceh, T	15	1	39
						-9	-18	63
						-9	-11	45
						4	-28	60
						-25	-19	56
						-5	-40	58
						-5	-47	-10
						-15	-16	5
						21	-20	5
		10			Rest > Backward Speech, 3 Y	-28	-81	-6

						-28	-84	9
						-14	-20	9
						16	-20	14
	Sachdev, 2008	10			Rest > Not Self, Normals	-10	50	-4
						-22	28	40
	Kerr, 2004	14			Brain Deactivation During R	-6	-53	44
						11	-55	48
						28	-44	68
						-23	-49	-11
						61	-25	39
						-18	-64	21
						16	-58	27
						-23	-63	-8
						-3	-90	28
						-9	-58	62
						-39	-79	31
						16	-55	66
						17	23	-8
						49	-74	34
						7	37	-13
						-18	-86	45
						-16	28	-16
						25	-84	45
						4	49	-24
						3	12	-11
						22	31	-25
	Shamosh, 2008	103			3-Back > Fixation, Deactivat	-3	40	7
						39	-9	-11
						-9	-62	12
	Gusnard, 2001	24			Fixation > Internally Cued C	4	37	-8
						-13	31	-1
						-7	28	-7
						8	15	-11
						-4	4	-14
					Fixation > Externally Cued C	4	37	-8
						-9	33	-3
						-2	28	-10
						8	17	-15

						0	8	-19
	Kelley, 2002	21			Fixation > All Trials	10	52	2
						-8	54	2
						6	40	44
						-4	4	-10
						18	34	52
						34	26	46
						14	58	28
						-14	50	26
						-24	36	-10
						12	-48	50
						-56	-58	38
						-34	-40	-12
						-16	-84	18
						-44	-76	30
						24	-8	-18
						56	-24	-14
	Liu, 2009	28			GB37 Acupuncture > Rest (D	-50	11	-3
						-18	-72	12
						3	-78	12
						-12	-80	26
						21	-55	3
						-30	-83	26
						12	-86	35
		28			KI8 Acupuncture > Rest (Dea	-3	55	0
						3	-18	48
						9	41	3
						23	26	-10
		28			K18 Acupuncture > GB37 Ac	-42	-15	-4
						42	-14	12
						-33	-15	-7
						30	-12	-15
	Binder, 2005	24			Fixation > Nonword	-57	-66	37
						-47	-72	40
						50	-58	27
						46	-74	42
						62	-65	32
						-25	26	43

						-21	58	7
						-36	20	41
						-20	36	46
						-29	53	-6
						30	54	0
						25	30	38
						20	60	4
						44	17	47
						19	50	-3
						37	27	55
						-31	10	-25
						-12	38	-5
						-8	23	-13
						37	45	-11
						-6	-54	39
						11	-58	26
						7	-43	40
						-31	-40	-3
						-35	15	-35
						-21	-21	25
						-22	-38	23
						-10	-68	27
	Boecker, 1998	7			Rest > Weighted Mean Perf	43	-82	16
						51	-64	23
						49	-73	2
						-42	-69	30
						-57	-39	0
						-26	-83	35
						-12	59	12
						1	68	-2
						-14	40	41
						-38	46	-8
						-16	36	-3
						-9	-60	28
						8	-62	24
						-55	-19	-11
	Jancke, 2000	8			Rest > Auditory Continuatio	60	-20	4
					Rest > Visual Continuation	-12	-88	0

						4	-80	4
