# Supplementary material for: Fusion and Fission of Cognitive Functions in the Human Parietal Cortex
Source: Cereb Cortex. 2014 Sep 9;25(10):3547–60. doi: 10.1093/cercor/bhu198 (PMC4585503; doi:10.1093/cercor/bhu198)
Supplement: Supplementary Data [file supp_bhu198_bhu198supp.docx]

Supplementary Material

**1a. Definitions of each ‘behavioural domain’**

In defining the behavioural domains and sub-categories included in the meta-analysis we sought to directly follow distinctions set up in the literature. As a result, we then minimise the influence of our own biases and ensure that the current results can be related *directly* to the existing literature.

***Attention:*** we differentiated between top-down attention and bottom-up attention, using the definitions given in prominent attention models and many reviews/meta-analyses ([Corbetta and Shulman 2002](#_ENREF_11); [Cabeza 2008](#_ENREF_5); [Ciaramelli et al. 2008](#_ENREF_10); [Hutchinson et al. 2009](#_ENREF_15); [Ciaramelli et al. 2010](#_ENREF_9); [Cabeza et al. 2012](#_ENREF_6)). Specifically, bottom-up attention involves reflexive and stimulus/driven attentional processes (e.g., automatic attentional capture during oddball tasks) and top-down attention involves preparing and applying goal-directed selection processes (e.g., cue period > target period in the Posner cueing task).

***Episodic retrieval***: was defined in a similar way to many existing reviews/meta-analyses, i.e., tasks that typically involve determining whether or not an item was previously seen or entirely new, and also correlations with the amount of episodic information that can be recalled for an item ([Cabeza 2008](#_ENREF_5); [Ciaramelli *et al.* 2008](#_ENREF_10); [Vilberg and Rugg 2008](#_ENREF_28); [Hutchinson *et al.* 2009](#_ENREF_15); [Ciaramelli *et al.* 2010](#_ENREF_9); [Kim 2010](#_ENREF_21); [Cabeza et al. 2011](#_ENREF_7); [Cabeza *et al.* 2012](#_ENREF_6)).

***Numerical processing***: researchers have distinguished between numerical calculation and fact retrieval ([Arsalidou and Taylor 2011](#_ENREF_1)). In numerical calculation, the solution to the problem cannot be retrieved from memory, rather it must be calculated during the task, such as in the case of large digit multiplication (e.g., 24 × 13). These tasks carry a high executive component. In contrast, numerical fact retrieval involves mathematical problems in which the solution can be automatically retrieved from memory, as in the case of small digit multiplication (e.g., 2 × 3).

***Linguistic tasks (semantics, phonology and sentence-level processing):*** we distinguished between semantic, phonological and sentence-level processing as evidence strongly suggests that these tasks engage distinct neural and psychological mechanisms ([Vigneau et al. 2006](#_ENREF_27)). Semantic processing was sub-categorised into automatic semantics and executive semantics (semantic control), which are known to differ ([Jefferies and Lambon Ralph 2006](#_ENREF_18)). Automatic semantics included word-level contrasts in which a semantic retrieval task was compared to a task of at least comparable difficulty and a comparison of semantically-rich over impoverished stimuli (e.g., concrete vs. abstract) within the same behavioural task such as lexical decision or reading ([Binder et al. 2009](#_ENREF_4)). Accordingly, the difficulty in activating the semantic concept is equal to/ lower than the item with which it is contrasted, e.g., concrete>abstract words. In contrast, "executive semantics" (or semantic control) included word-level studies which involve high- compared to low-semantic control demands, such as the distinction between ambiguous>unambiguous words. Sentence-level processing included contrasts of any task which involved increased demands on combinatorial/syntactic processing at the sentence level (e.g., high syntactic complexity > low complexity) as these processes have been shown to be partially distinct from word-level processing ([Vigneau *et al.* 2006](#_ENREF_27)). Phonological processing refers to any manipulation or decision about the phonological form of words (e.g., rhyme or syllable judgements).

***Tool-praxis decisions:*** included any study involving recognition, naming, or action-related (praxis) decisions related to tools ([as defined in Ishibashi et al. submitted](#_ENREF_16)).

***DMN:*** was defined using the Brainmap database (<http://www.brainmap.org/>). We selected all studies (from any behavioural domain) in the database that showed task-related deactivations relative to a low level control condition (either fixation or rest) using healthy participants. This is considered a robust and unbiased method for identifying the DMN and has been used successfully elsewhere in the literature ([Laird et al. 2009](#_ENREF_22); [Smith et al. 2009](#_ENREF_26)).

**1b. Control analyses for study-selection biases**

Given that we primarily adopted selection criteria from previous single-domain meta-analyses and review, additional analyses were performed to assess the impact of variation in selection criteria on the results. In particular we explored whether studies adopted a whole-brain or an ROI based approach to their analyses, or whether they contrasted with a low-level baseline condition (rest/fixation). To examine the influence of these factors, the primary ALE analysis was re-run excluding cases using an ROI-based correction method (which was very few - typically one or two studies per domain) and found that this had a negligible impact on the parietal results with only very minor changes in the extent (but not location) of some clusters. We also examined the influence of including studies using only a low-level baseline condition (rest or fixation). Across all domains contributing to the parietal results, only eight involved a contrast with a low-level baseline condition (fixation or rest). Seven of these studies were from the domain of top-down attention (the remaining study was from the bottom-up attention domain). Repeating the analyses with these studies excluded had little effect on the location of the clusters, although for "top-down attention" there was a reduction in the extent of the dorsal right hemisphere (but not left hemisphere) parietal cluster likely due to a reduction in statistical power.

**1c. Results of the meta-analysis for non-parietal regions**

Although not directly relevant to the aims of the main study, the results from the ALE analysis also revealed results from non-parietal areas that have bearing on current issues in the language and semantics literatures. Firstly, there is ongoing discussion in the language literature with regard specialisation of lateral prefrontal cortex for certain linguistic tasks. For instance, it has been suggested that phonology, syntax and semantics engage distinct areas of left inferior front al gyrus (LIFG), with a posterior-anterior gradient of function ([Gough et al. 2005](#_ENREF_12); [Hagoort 2005](#_ENREF_13); [Xiang et al. 2010](#_ENREF_30)). The results from the current meta-analysis provide some support for a neuroanatomically-graded variant of this hypothesis. All three tasks engaged a common region of middle frontal gyrus. However, within LIFG phonological decisions were found to recruit more posterior areas of prefrontal cortex, including pars opercularis (BA44) and premotor cortex (BA6). In contrast, both semantic decisions and sentence level processing (which included both semantic and syntactic contrasts) engaged overlapping areas of pars triangularis (BA45) and pars orbitalis (BA47). These results are depicted in Figure S1 (left panel). In summary, these results suggest that all three language tasks engage a common middle prefrontal cortex region and then diverge within LIFG, with phonological decisions recruiting additional posterior areas, whilst semantic and syntactic processes spread anteriorly.

In terms of semantic processes in the temporal lobe, there are ongoing questions regarding the function of the posterior middle temporal gyrus (pMTG). Given the proximity of pMTG to visual motion processing area MT, it has been argued that pMTG stores semantic knowledge related to motion ([Chao et al. 1999](#_ENREF_8); [Martin and Chao 2001](#_ENREF_23); [Beauchamp et al. 2002](#_ENREF_2), [2003](#_ENREF_3)). Indeed, pMTG has been shown to be activated by semantic tasks that involve a manipulation of motion properties, such as contrasts involving semantic decision made on tools (which possess strong motion features) compared to non-motion categories ([Martin et al. 1996](#_ENREF_24); [Kable et al. 2005](#_ENREF_19)), decisions made on action verbs compared to nouns ([Kable et al. 2002](#_ENREF_20); [Kable *et al.* 2005](#_ENREF_19)), and also when listening to sentences that describe motion compared to sentences without motion ([Humphreys et al. 2013](#_ENREF_14)). However, there are parallel results which suggest that pMTG is not specialised for semantic representation but rather it forms a central component of the semantic control network – an executive system that acts upon a wide variety of semantic information in order to develop context appropriate behaviour ([Jefferies and Lambon Ralph 2006](#_ENREF_18); [Jefferies 2013](#_ENREF_17)). Indeed, a meta-analysis of semantic control studies finds reliable pMTG activation ([Noonan et al. 2013](#_ENREF_25)) and rTMS of pMTG impairs executively-demanding semantic tasks to a relatively greater degree ([Whitney et al. 2012](#_ENREF_29)). By undertaking a simultaneous direct comparison, the current study helps resolve this apparent discrepancy: partially-overlapping areas of pMTG are involved in tool-related tasks (i.e., motion tasks) compared to semantic ‘control’ (see Figure S2, right panel). Specifically, tool-related tasks engaged more posterior areas of pMTG, with partial overlap across tasks. Thus, it appears that pMTG is not a homogeneous area and that there is an important posterior-anterior shift in pMTG function.

**1d. Polarity of activation relative to rest/fixation:**

We examined the polarity of activation (positive or negative) relative to rest/fixation from those studies that contributed foci to each of the parietal clusters in order to determine any systematic variations across regions/domains. Studies were excluded from this analysis if they failed to report the results of a contrast of task > rest/fixation (the percentages of studies that reported this information can be seen in Tables S3-5).

Supplementary references

Arsalidou M, Taylor MJ. 2011. Is 2+2=4? Meta-analyses of brain areas needed for numbers and calculations. NeuroImage 54:2382-2393.

Beauchamp MS, Lee KE, Haxby JV, Martin A. 2002. Parallel visual motion processing streams for manipulable objects and human movements. Neuron 34:149-159.

Beauchamp MS, Lee KE, Haxby JV, Martin A. 2003. FMRI responses to video and point-light displays of moving humans and manipulable objects. J Cogn Neurosci 15:991-1001.

Binder JR, Desai RH, Graves WW, Conant L. 2009. Where Is the Semantic System? A Critical Review and Meta-Analysis of 120 Functional Neuroimaging Studies. Cereb Cortex 19:2767-2796.

Cabeza R. 2008. Role of parietal regions in episodic memory retrieval: The dual attentional processes hypothesis. Neuropsychologia 46:1813-1827.

Cabeza R, Ciaramelli E, Moscovitch M. 2012. Cognitive contributions of the ventral parietal cortex: an integrative theoretical account. Trends in Cognitive Sciences 16:338-352.

Cabeza R, Mazuz YS, Stokes J, Kragel JE, Woldorff MG, Ciaramelli E, Olson IR, Moscovitch M. 2011. Overlapping Parietal Activity in Memory and Perception: Evidence for the Attention to Memory Model. Journal of Cognitive Neuroscience 23:3209-3217.

Chao LL, Haxby JV, Martin A. 1999. Attribute-based neural substrates in temporal cortex for perceiving and knowing about objects. Nat Neurosci 2:913-919.

Ciaramelli E, Grady C, Levine B, Ween J, Moscovitch M. 2010. Top-Down and Bottom-Up Attention to Memory Are Dissociated in Posterior Parietal Cortex: Neuroimaging and Neuropsychological Evidence. The Journal of neuroscience : the official journal of the Society for Neuroscience 30:4943-4956.

Ciaramelli E, Grady CL, Moscovitch M. 2008. Top-down and bottom-up attention to memory: A hypothesis (AtoM) on the role of the posterior parietal cortex in memory retrieval. Neuropsychologia 46:1828-1851.

Corbetta M, Shulman GL. 2002. Control of goal-directed and stimulus-driven attention in the brain. Nat Rev Neurosci 3:201-215.

Gough PM, Nobre AC, Devlin JT. 2005. Dissociating linguistic processes in the left inferior frontal cortex with transcranial magnetic stimulation. The Journal of neuroscience : the official journal of the Society for Neuroscience 25:8010-8016.

Hagoort P. 2005. On Broca, brain, and binding: a new framework. Trends in Cognitive Sciences 9:416-423.

Humphreys GF, Newling K, Jennings C, Gennari SP. 2013. Motion and actions in language: semantic representations in occipito-temporal cortex. Brain and Language 125:94-105.

Hutchinson JB, Uncapher MR, Wagner AD. 2009. Posterior parietal cortex and episodic retrieval: Convergent and divergent effects of attention and memory. Learn Memory 16:343-356.

Ishibashi R, Pobric G, Saito S, Lambon Ralph MA. submitted. The neural network for tool-related cognition: An activation likelihood estimation meta-analysis of 49 neuroimaging studies.

Jefferies E. 2013. The neural basis of semantic cognition: converging evidence from neuropsychology, neuroimaging and TMS. Cortex 49:611-625.

Jefferies E, Lambon Ralph MA. 2006. Semantic impairment in stroke aphasia versus semantic dementia: a case-series comparison. Brain 129:2132-2147.

Kable JW, Kan IP, Wilson A, Thompson-Schill SL, Chatterjee A. 2005. Conceptual representations of action in the lateral temporal cortex. J Cogn Neurosci 17:1855-1870.

Kable JW, Lease-Spellmeyer J, Chatterjee A. 2002. Neural substrates of action event knowledge. J Cogn Neurosci 14:795-805.

Kim H. 2010. Dissociating the roles of the default-mode, dorsal, and ventral networks in episodic memory retrieval. NeuroImage 50:1648-1657.

Laird AR, Eickhoff SB, Li K, Robin DA, Glahn DC, Fox PT. 2009. Investigating the functional heterogeneity of the default mode network using coordinate-based meta-analytic modeling. The Journal of neuroscience : the official journal of the Society for Neuroscience 29:14496-14505.

Martin A, Chao LL. 2001. Semantic memory and the brain: structure and processes. Current Opinion in Neurobiology 11:194-201.

Martin A, Wiggs CL, Ungerleider LG, Haxby JV. 1996. Neural correlates of category-specific knowledge. Nature 379:649-652.

Noonan KA, Jefferies E, Lambon Ralph MA. 2013. Going beyond inferior prefrontal involvement in semantic control: Evidence for the additional contribution of parietal and posterior middle temporal cortex. Journal of Cognitive Neuroscience 25:1824-1850.

Smith SM, Fox PT, Miller KL, Glahn DC, Fox PM, Mackay CE, Filippini N, Watkins KE, Toro R, Laird AR, Beckmann CF. 2009. Correspondence of the brain's functional architecture during activation and rest. Proc Natl Acad Sci U S A 106:13040-13045.

Vigneau M, Beaucousin V, Herve PY, Duffau H, Crivello F, Houde O, Mazoyer B, Tzourio-Mazoyer N. 2006. Meta-analyzing left hemisphere language areas: Phonology, semantics, and sentence processing. NeuroImage 30:1414-1432.

Vilberg KL, Rugg MD. 2008. Memory retrieval and the parietal cortex: A review of evidence from a dual-process perspective. Neuropsychologia 46:1787-1799.

Whitney C, Kirk M, O'Sullivan J, Lambon Ralph MA, Jefferies E. 2012. Executive Semantic Processing Is Underpinned by a Large-scale Neural Network: Revealing the Contribution of Left Prefrontal, Posterior Temporal, and Parietal Cortex to Controlled Retrieval and Selection Using TMS. Journal of Cognitive Neuroscience 24:133-147.

Xiang HD, Fonteijn HM, Norris DG, Hagoort P. 2010. Topographical functional connectivity pattern in the perisylvian language networks. Cereb Cortex 20:549-560.

**Supplementary Tables:**

Table S1. A summary of all included studies (excel file).

Table S2. Parietal activation clusters for each task derived from the primary ALE analysis.

| Task | Cluster # | Volume (mm³) | x | y | z | Location | BA |
| --- | --- | --- | --- | --- | --- | --- | --- |
| Automatic Semantics | 1 | 8784 | -48 | -68 | 28 | Left angular gyrus | 39 |
|  |  |  | -34 | -72 | 42 | Left precuneus | 19 |
|  | 2 | 8160 | -4 | -56 | 12 | Left posterior cingulate | 29 |
|  |  |  | -8 | -50 | 36 | Left precuneus | 31 |
|  |  |  | 10 | -50 | 36 | Right precuneus | 31 |
|  |  |  | 10 | -52 | 24 | Right posterior cingulate | 31 |
|  | 3 | 2640 | 46 | -70 | 38 | Right precuneus | 39 |
|  |  |  | 44 | -60 | 40 | Right angular gyrus | 39 |
|  | 4 | 200 | 54 | -48 | 34 | Right supramarginal gyrus | 40 |
| Bottom-up Attention | 1 | 2512 | 56 | -42 | 14 | Right supramarginal gyrus | 40 |
|  | 2 | 1504 | -26 | -62 | 56 | Left precuneus | 7 |
|  | 3 | 1200 | 36 | -48 | 54 | Right superior parietal lobule | 7 |
|  |  |  | 38 | -42 | 44 | Right supramarginal gyrus | 40 |
|  | 4 | 600 | -62 | -46 | 18 | Left supramarginal gyrus | 40 |
|  | 5 | 496 | 32 | -70 | 36 | Right precuneus | 19 |
|  | 6 | 408 | -26 | -70 | 26 | Left precuneus | 31 |
|  | 7 | 312 | 6 | -58 | 52 | Right precuneus | 7 |
|  | 8 | 168 | 18 | -68 | 44 | Right precuneus | 7 |
| Default-Mode Network | 1 | 28224 | -2 | -56 | 50 | Left superior parietal lobule | 7 |
|  |  |  | -4 | -52 | 24 | Left precuneus | 23 |
|  |  |  | -2 | -72 | 32 | Left precuneus | 31 |
|  |  |  | 4 | -46 | 48 | Right precuneus | 7 |
|  |  |  | 10 | -26 | 40 | Right cingulate gyrus | 31 |
|  | 2 | 10552 | 50 | -68 | 20 | Right angular gyrus | 39 |
|  | 3 | 2512 | -44 | -68 | 24 | Left angular gyrus | 39 |
|  | 4 | 2064 | -60 | -34 | 30 | Left supramarginal gyrus | 40 |
|  |  |  | 58 | -26 | 24 | Right supramarginal gyrus | 40 |
| Episodic Retrieval | 1 | 8624 | -40 | -70 | 38 | Left angular gyrus | 39 |
|  |  |  | -48 | -54 | 48 | Left IPS | 7 |
|  | 2 | 2736 | -2 | -70 | 38 | Left precuneus | 7 |
|  |  |  | -4 | -56 | 30 | Left cingulate gyrus | 31 |
|  | 3 | 2672 | 40 | -54 | 44 | Right precuneus | 19 |
| Numerical calculation | 1 | 4416 | -48 | -38 | 42 | Left IPS | 7 |
|  |  |  | -34 | -56 | 58 | Left superior parietal lobule | 7 |
|  | 2 | 2680 | 36 | -60 | 50 | Right superior parietal lobule | 7 |
|  | 3 | 1800 | 44 | -42 | 44 | Right IPS | 7 |
| Numerical Fact Retrieval | 1 | 4416 | -50 | -60 | 30 | Left angular gyrus | 39 |
|  | 2 | 2192 | -8 | -56 | 24 | Left cingulate gyrus | 31 |
|  | 3 | 776 | 24 | -80 | 34 | Right cuneus | 18 |
|  | 4 | 312 | 6 | -64 | 60 | Right precuneus | 7 |
| Phonological Processing | 1 | 8768 | -42 | -42 | 40 | Left IPS | 7 |
|  |  |  | -28 | -60 | 42 | Left precuneus | 19 |
|  |  |  | -32 | -54 | 50 | Left superior parietal lobule | 7 |
|  | 2 | 544 | 32 | -60 | 42 | Right IPS | 7 |
|  | 3 | 280 | -56 | -42 | 24 | Left supramarginal gyrus | 40 |
| Top-down Attention | 1 | 13672 | -30 | -50 | 48 | Left superior parietal lobule | 7 |
|  |  |  | -26 | -66 | 40 | Left IPS | 7 |
|  |  |  | -24 | -56 | 62 | Left precuneus | 7 |
|  | 2 | 3848 | 30 | -52 | 54 | Right superior parietal lobule | 7 |
|  | 3 | 936 | 34 | -72 | 26 | Right precuneus | 31 |
| Top-down Semantics | 1 | 336 | -38 | -46 | 50 | Left IPS | 7 |
| Tools | 1 | 6440 | -42 | -32 | 46 | Left postcentral gyrus | 2 |
|  |  |  | -32 | -42 | 48 | Left IPS | 7 |
|  |  |  | -36 | -42 | 62 | Left superior parietal lobule | 7 |
|  |  |  | -60 | -28 | 40 | Left supramarginal gyrus | 40 |
|  | 2 | 656 | -26 | -52 | 72 | Left superior parietal lobule | 7 |
|  | 3 | 264 | -26 | -74 | 38 | Left precuneus | 19 |

**Table S3 The percentage to report a contrast with rest from the foci that contributed to the AG cluster**

| AG |  |  |  |  |
| --- | --- | --- | --- | --- |
| Contrast | Automatic semantics | Episodic retrieval | Numerical fact retrieval | Sentence-level |
| Percentage reporting contrast with rest/fixation | 27% | 55% | 60% | 60% |

**Table S4 The percentage to report a contrast with rest from the foci that contributed to the SMG cluster**

| SMG |  |  |
| --- | --- | --- |
| Contrast | Bottom-up attention | Phonology |
| Percentage reporting contrast with rest/fixation | 75% | 0% |

**Table S5 The percentage to report a contrast with rest from the foci that contributed to the IPS/SPL cluster**

| IPS/SPL |  |  |  |  |  |
| --- | --- | --- | --- | --- | --- |
| Contrast | Top-down attention | Numerical calculation | Executive semantics | Tools | Phonology |
| Percentage reporting contrast with rest/fixation | 91% | 20% | 100% | 76% | 11% |

**Supplementary Figures:**

Figure S1. The results from the ALE Subtraction Analysis 1, contrasting all dorsal and ventral tasks.

Figure S2. Non-parietal results from the ALE analysis. The left panel shows the results from phonological, top-down semantic, and sentence level tasks within the prefrontal cortex. The right panel shows the results from top-down semantics and tool-related tasks in the pMTG.

Figure S3. The results from the whole-brain ALE analysis. Meta-analysis results were thresholded at FDR correction of p < .05 and a minimum cluster size of 100mm³.
