# Supplementary figures and images for: Fusion and Fission of Cognitive Functions in the Human Parietal Cortex
Source: Cereb Cortex. 2014 Sep 9;25(10):3547–60. doi: 10.1093/cercor/bhu198 (PMC4585503; doi:10.1093/cercor/bhu198)

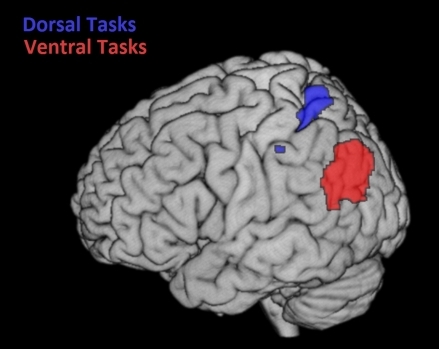

Supplement: Supplementary Data [file supp_bhu198_bhu198supp_fig1.jpg]

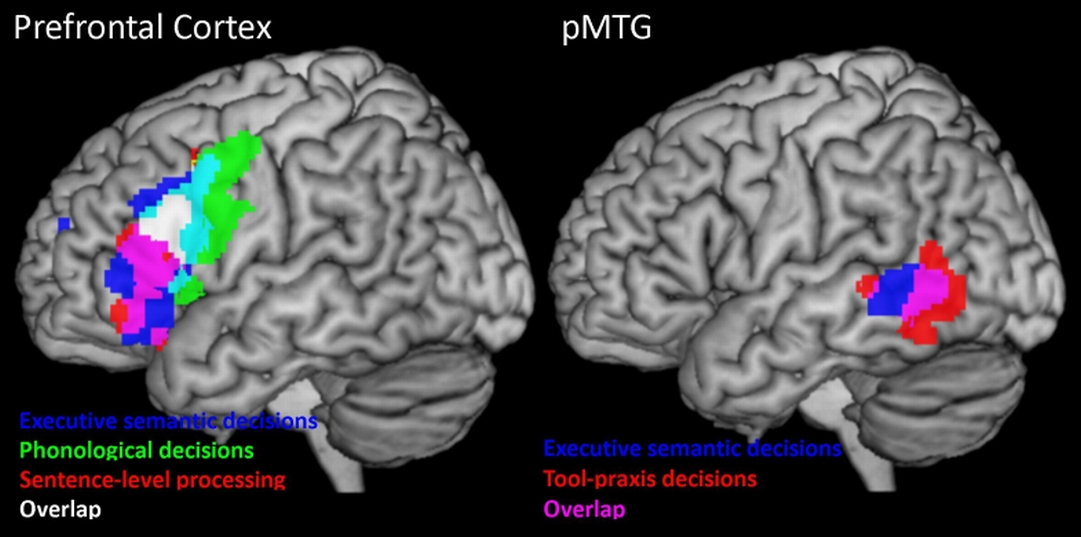

Supplement: Supplementary Data [file supp_bhu198_bhu198supp_fig2.jpg]

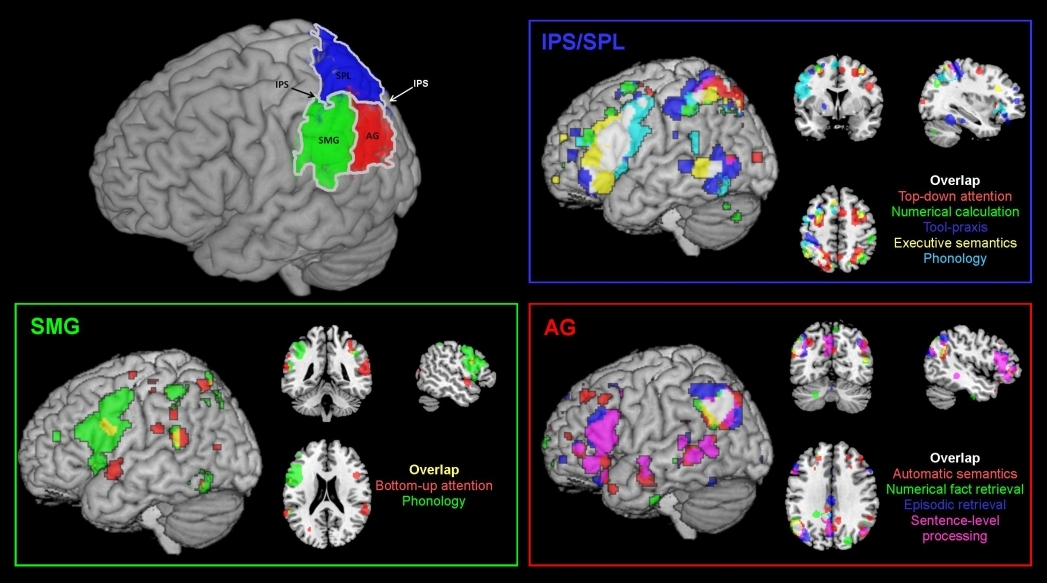

Supplement: Supplementary Data [file supp_bhu198_bhu198supp_fig3.jpg]
